# Radiotherapy-Conditioned Antibody–Drug Conjugate Activity: Molecular Mechanisms, Therapeutic Windows and Organ-Specific Toxicity

**DOI:** 10.3390/cancers18213554

**Published:** 2026-11-05

**Authors:** Ingrid C. Landfald, Łukasz Olewnik, Magdalena Ciechanowska, Maciej Kornatowski

**Affiliations:** 1 Department of Clinical Anatomy, Mazovian University in Płock, 09-402 Płock, Poland; 2 VARIA Research Laboratory, 09-402 Płock, Poland; 3 VARIANTIS Research Laboratory, 09-402 Płock, Poland; 4 Faculty of Health Sciences, Mazovian University in Płock, 09-402 Płock, Poland; m.ciechanowska@mazowiecka.edu.pl; 5 Faculty of Medicine, Collegium Medicum, Mazovian University in Płock, 09-402 Płock, Poland; m.kornatowski@mazowiecka.edu.pl

**Keywords:** antibody–drug conjugates, radiotherapy, radiosensitization, target plasticity, payload release, tumor microenvironment, antitumor immunity, treatment sequencing, radionecrosis

## Abstract

Antibody–drug conjugate (ADC) activity depends on accessible targets, tissue distribution, payload release, cellular susceptibility, and host immunity. Radiotherapy (RT) can modify these determinants, creating a time- and tissue-dependent radiation-conditioned ADC state. This critical narrative review integrates mechanistic and clinical evidence, distinguishing intact ADC–RT experiments from studies of isolated components, ADCs without RT, and engineered platforms. Direct preclinical studies support selected target-conditioning, payload, and immune interactions, but do not establish a class-wide effect. We organize the evidence into four overlapping, potentially competing biological windows: targeting, radiosensitization, immune priming, and normal-tissue injury. The framework links mechanisms to schedule comparisons, measurements, and organ-specific constraints. Delivery-mediated interactions require altered exposure; response-mediated effects may occur at comparable exposure. Clinical evidence is predominantly observational and heterogeneous in the drug, irradiated site, fractionation, overlap, and follow-up. Feasibility reports involving trastuzumab deruxtecan and the association between trastuzumab emtansine and radionecrosis after stereotactic radiosurgery require a separate interpretation. Biological susceptibility also differs from later injury detection. Prospective studies should compare durable tumor control, treatment continuity, and acute and delayed toxicity, using selected mechanistic substudies. The framework is hypothesis-generating and does not establish a universal treatment sequence.

## Simple Summary

Antibody–drug conjugates (ADCs) deliver potent drugs to cells that carry a recognized target. Many patients receiving these treatments also need radiation, but the best way to combine them is uncertain. Radiation can change how a drug reaches a tumor, how cancer cells respond to its payload, and how immune cells and healthy tissues respond to treatment. These changes may occur at different times and can have competing effects. This review brings together laboratory and clinical findings using four biological windows: targeting, radiosensitization, immune priming, and normal-tissue injury. The framework helps identify which interaction a study should test and which measurements are needed. It does not prescribe a treatment sequence. Future studies should compare schedules for specific drugs and organs, measure tumor control and treatment completion, and follow patients long enough to detect delayed injury.

## 1. Introduction

Antibody–drug conjugates (ADCs) combine a target-directed antibody with a therapeutic payload, usually a potent cytotoxic agent. Their activity depends on more than antigen recognition: target accessibility, tissue penetration, internalization, linker processing, payload release, and cellular susceptibility all influence the response. Antibody effector functions and immune interactions may contribute, while off-tumor uptake and systemic payload exposure constrain tolerability [1,2].

Radiotherapy (RT) can change several of these determinants. Experiments have demonstrated changes in selected surface antigens, vascular characteristics, and antigen presentation after irradiation [3,4,5]. These findings justify testing ADC–RT combinations, but each measures a different process. Throughout this review, we distinguish intact ADC–RT experiments from ADC studies without RT and from indirect evidence involving unconjugated antibodies, free payloads, or engineered conjugates.

Direct combination studies provide more specific support. Lewis et al. investigated an experimental ADC directed against tax-interacting protein 1 (TIP1) and carrying monomethyl auristatin E (MMAE); treatment enhanced the radiation response in lung-cancer models [6]. Hingorani et al. demonstrated functional CD8-positive T-cell contributions and enhanced the checkpoint-blockade activity in selected auristatin-conjugate/RT models [7]. The immune consequences of irradiation can also be unfavorable: the DNA exonuclease three-prime repair exonuclease 1 (TREX1) can suppress radiation-induced innate signaling, and tumor-antigen-specific T cells may be preferentially depleted in some clinical settings [8,9].

Clinical findings likewise depend on the drug and treatment setting. TENDANCE reported the feasibility of trastuzumab deruxtecan (T-DXd) with RT in a selected observational cohort [10]. In contrast, trastuzumab emtansine (T-DM1) exposure has been associated with clinically significant radionecrosis after stereotactic radiosurgery (SRS) [11]. These observations support drug-, organ-, and schedule-specific assessment.

Previous reviews have addressed ADC-mediated radiosensitization and the rationale for combining ADCs, RT, and immunotherapy [12,13]. Our contribution is an operational synthesis: a proposed mechanism is connected to the treatment interval it might favor, the competing normal-tissue or immune effects, and the measurements needed to compare schedules. We use the radiation-conditioned ADC state to describe the time- and tissue-dependent conditions under which an ADC acts after irradiation, rather than an assumed change in the drug itself.

We organize these conditions into four windows: targeting, radiosensitization, immune priming, and normal-tissue injury. Unlike a fixed sequence, the framework allows beneficial and harmful processes to overlap. A worked preclinical example and separate applications to T-DM1, T-DXd, and sacituzumab govitecan (SG) show how it can generate testable questions. It remains a hypothesis-generating tool, with no validated window boundaries or universal withholding interval.

## 2. Literature-Search Approach and Scope

This article is a critical narrative review of the molecular, translational, and clinical evidence concerning interactions between ADCs and RT. The review was designed to evaluate the mechanistic plausibility, strength of evidence, clinical feasibility, and organ-specific toxicity rather than to estimate a pooled treatment effect; it is therefore not a systematic review or meta-analysis.

PubMed, including MEDLINE-indexed records, and Scopus were searched from inception through 1 October 2026. Search concepts combined antibody–drug conjugate (ADC) class terms and individual agent names with radiotherapy (RT), radiation, irradiation, radiosensitization, stereotactic radiosurgery (SRS), and stereotactic body radiotherapy (SBRT). Reference-list screening and forward-citation searching supplemented database retrieval. All studies identified through these approaches and considered relevant to the review’s mechanistic, translational, or clinical scope were eligible for inclusion. Supplementary Methods S1 presents database-specific search formulations reconstructed from the search concepts and lists the agents represented in the synthesis; these formulations are not archived copies of the original queries.

ClinicalTrials.gov was searched on 1 October 2026 to identify relevant prospective studies. Registry entries, published protocols, conference abstracts, and full publications were distinguished according to their source and reporting stage. Secondary analyses were interpreted in relation to their parent studies rather than treated as independent cohorts. Where sources differed in protocol details or participant populations, these differences were explicitly acknowledged. Selected prospective studies and their principal design questions are summarized in Section 14.5 without making claims regarding the current recruitment status.

Evidence was eligible if it addressed target availability, drug delivery or processing, payload–radiation response, immunity, resistance, clinical outcomes, toxicity, or prospective trial design. Intact ADC–RT studies were prioritized for combination-specific conclusions. Indirect experiments were retained when they informed a defined mechanism and are labeled by the material actually tested. The evidence tables selectively summarize mechanistic findings, clinical reports across distinct drugs and treatment settings, and prospective protocols addressing different sequencing questions; none is intended as an exhaustive evidence inventory.

Claims were cross-matched with the primary reports available for examination, including experimental arms, denominators, timing, and endpoint definitions. Supplementary Tables S1 and S2 separate study conditions from interpretation and distinguish directly measured exposure from imaging or pharmacodynamic surrogates. Source limitations are described explicitly in the supplementary tables. Information unavailable in an examined abstract must not be interpreted as absent from the full publication; it is distinguished from information not reported in an examined full text.

No pooled analysis or formal risk-of-bias instrument was used. Interpretation considers selection, confounding, treatment heterogeneity, repeated lesions within patients, competing mortality, and observation time. Conference abstracts and protocols are identified explicitly and are not counted as independent confirmation of their parent study. Evidence syntheses may reuse the original cohorts discussed here and are not additional patient series. Feasibility means treatment was deliverable under the reported conditions; synergy requires an appropriate interaction analysis.

## 3. A Functional Framework for ADC–RT Interactions

**Figure 1:**
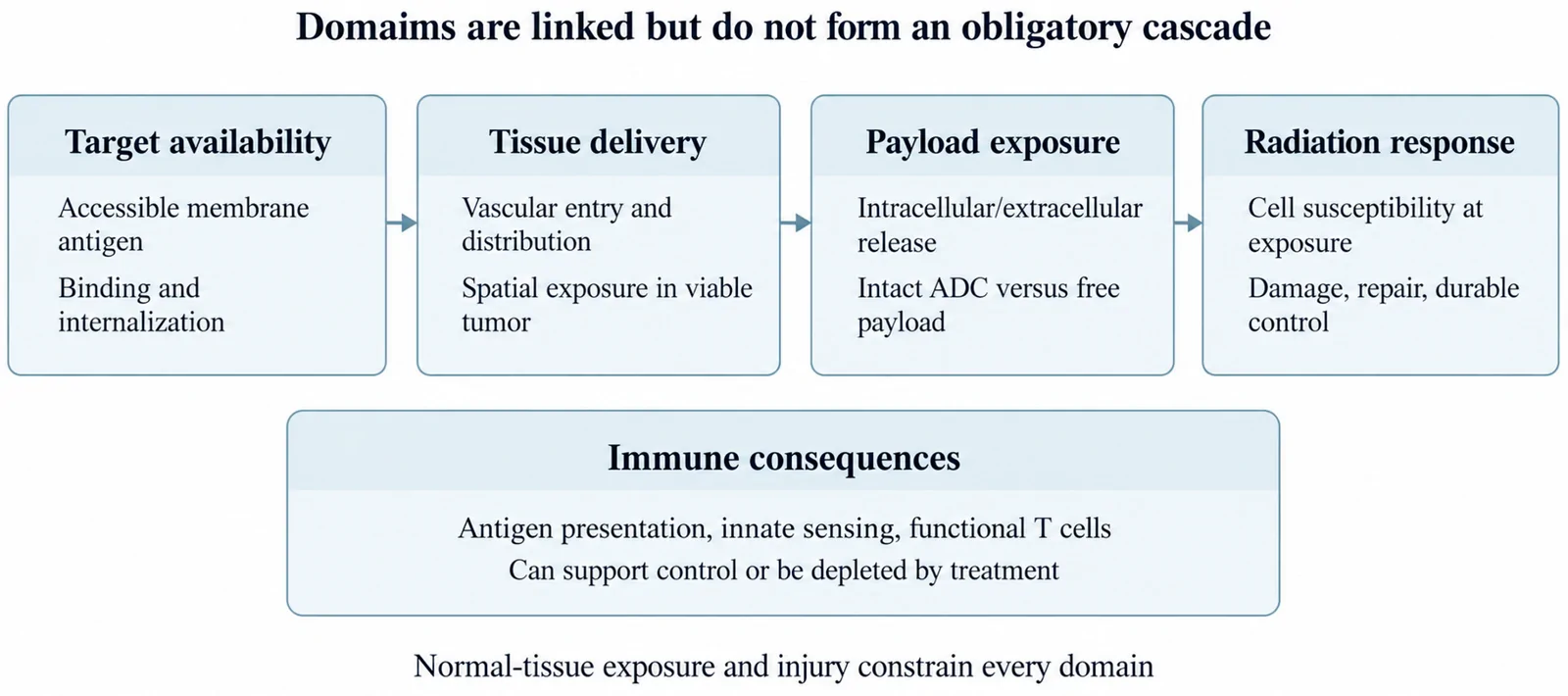
Functional domains governing interactions between antibody–drug conjugates and radiotherapy. The framework separates target availability, tumor delivery, payload release, radiation response, and immune consequences as distinct mechanistic questions. Each domain requires direct assessment, while normal-tissue exposure and toxicity should be evaluated across the entire treatment pathway. ADC, antibody–drug conjugate.

An ADC–RT hypothesis should specify which process irradiation is intended to modify. Five linked domains organize the mechanistic evidence: target availability and binding; tissue delivery and distribution; payload release and exposure; cellular radiation response; and immune consequences (Figure 1).

These are analytical domains, not obligatory steps. Extracellular cleavage may bypass tumor-cell internalization, and direct cytotoxicity does not require an adaptive immune response. The study should identify the limiting process for the particular conjugate and test whether changing it improves tumor control at an acceptable cost in normal-tissue injury.

### 3.1. Target Availability and Spatial Delivery

Target expression must be interpreted together with antibody access. In preclinical studies without RT, transient competitive inhibition improved trastuzumab penetration and enhanced T-DM1 efficacy [14]. The result illustrates a distribution constraint; it does not demonstrate that irradiation overcomes it.

Radiation-induced target upregulation and improved ADC delivery should therefore be treated as distinct hypotheses. An increased surface-antigen abundance does not establish that more intact conjugate reaches viable tumor cells throughout the lesion. Likewise, radiation-induced changes in vascular imaging parameters do not, by themselves, demonstrate improved macromolecular delivery [4,5]. Studies evaluating target conditioning should therefore assess accessible antigen, functional binding, and the spatial distribution of the intact ADC in parallel.

### 3.2. Payload Release and Bystander Exposure

The released payload can reach cells that the antibody does not bind. Ogitani et al. demonstrated the bystander killing of neighboring human epidermal growth factor receptor 2 (HER2)-negative cells by T-DXd in mixed experimental systems without RT [15]. This local effect did not establish an equivalent exposure in distant tumors and should not be generalized to all ADC payloads.

Tsao et al. demonstrated extracellular T-DXd cleavage involving tumor-microenvironment proteases, including cathepsin L, in breast-cancer models without RT [16]. This establishes an additional release route. A claim that irradiation enhances that route would require paired measurements of the protease activity, linker cleavage, and released payload after RT.

### 3.3. Radiation Response, Immunity and Therapeutic Index

The active payload, cellular susceptibility, and the RT regimen jointly determine the response. Selected auristatin-conjugate studies demonstrate functional immune contributions to combined treatment [7], whereas TREX1 induction and lymphocyte depletion provide separate evidence for competing radiation effects [8,9]. These indirect constraints have not been validated as scheduling biomarkers for ADC–RT treatment.

The therapeutic-index question requires both durable tumor control and acute and delayed toxicity. Molecular activity is useful for explaining an interaction, but its clinical value depends on whether the gain outweighs harm in the irradiated organ.

### 3.4. Molecular Coupling Points and Causal Evidence

Potential coupling occurs at the target, processing, payload, or host level. Surface-antigen induction concerns binding; altered trafficking or protease activity concerns release; cell-cycle and repair changes concern the payload response; and antigen presentation or lymphocyte function concerns immunity. The cited experiments address different interfaces and should not be read as one continuous demonstration [4,7,8,16,17].

Two causal models should be distinguished. A delivery-mediated hypothesis predicts that RT changes access or processing, producing a change in the active drug exposure and then response. A response-mediated hypothesis predicts an altered radiosensitivity or immune function at a comparable exposure; increased exposure is not required. Exposure measurements help separate these explanations, while functional perturbations test the proposed mediator. Selected studies support parts of either model [6,7,17,18,19], but none validates a class-wide pathway.

## 4. RT-Induced Target Plasticity: When Does Antigen Modulation Become Therapeutically Useful?

### 4.1. Surface-Antigen Induction

Wattenberg et al. reported the radiation-associated induction of selected antibody targets, including HER2, epidermal growth factor receptor (EGFR), and CD20, and enhanced trastuzumab-mediated antibody-dependent cellular cytotoxicity [4]. These are unconjugated-antibody findings, not measurements of ADC payload delivery.

Lewis et al. directly tested a TIP1-directed MMAE ADC with RT [6]. Xing et al. tested an intercellular adhesion molecule 1 (ICAM1)-directed ADC carrying the topoisomerase-I inhibitor payload DXd [17]. In the latter study, ICAM1 induction after 2 Gy was followed over time, and combination treatment improved the control of established xenografts. Fluorescent unconjugated-antibody uptake and cellular internalization supported targeting, but did not quantify the released DXd. DXd denotes the payload; T-DXd denotes the complete HER2-directed ADC.

Target induction is useful only if it occurs in surviving, accessible cells and supports productive delivery. Normal-tissue selectivity also requires an appropriate model. The ICAM1 antibody in Xing et al. did not cross-react with mouse ICAM1, limiting conclusions about on-target normal-tissue toxicity; nude-mouse xenografts also cannot establish a functional adaptive immune contribution [17].

### 4.2. Antigen Presentation and ADC Recognition Are Distinct Mechanisms

RT can improve immune recognition without increasing the target bound by an ADC. Reits et al. demonstrated changes in the major histocompatibility complex class I (MHC-I) peptide repertoire after irradiation [3]; Gameiro et al. studied the immunogenic modulation of surviving tumor cells [20,21]. Neither constitutes direct evidence of enhanced ADC uptake.

This distinction is important when attributing combination effects. Improved tumor control may result from enhanced payload delivery, improved immune recognition of surviving cancer cells, or both. Tumor-volume measurements alone cannot distinguish these mechanisms. When improved ADC delivery is the proposed explanation, target quantification and direct measurements of ADC or payload exposure should therefore accompany immune analyses.

### 4.3. Heterogeneity Limits Expression-Based Scheduling

Baseline target positivity does not ensure that all viable disease remains uniformly targetable during treatment. Spatial heterogeneity is present even in the absence of RT. Sabtan et al. [22] characterized the MET proto-oncogene receptor tyrosine kinase (c-MET), nectin-4, and trophoblast cell-surface antigen 2 (TROP2) expression in pancreatic ductal adenocarcinoma and identified target-specific differences in spatial distribution and heterogeneity. These findings support the need for a spatial assessment, but they do not establish that irradiation induces antigen loss, lineage switching, or the selection of antigen-low clones. Such mechanisms require longitudinal evidence within the relevant ADC–RT setting.

Serial circulating tumor-cell analysis may provide complementary information, although its interpretation requires caution. Mishra et al. [23] found that changes in circulating tumor-cell numbers tracked the treatment response more closely than target-epitope expression. This challenges the assumption that persistent antigen positivity necessarily predicts continued ADC sensitivity. Resistance may emerge despite target retention, while circulating cells cannot directly establish target accessibility within an irradiated lesion.

Treatment monitoring should therefore distinguish among target abundance, target accessibility, and payload susceptibility. Repeated expression measurements can document biological change, but they do not, by themselves, identify which component limits the treatment response.

## 5. RT and Intratumoral ADC Access

The evidence that RT can alter antibody uptake is stronger than the evidence that it consistently improves intratumoral ADC distribution. Kalofonos et al. [24] demonstrated an increased uptake of radiolabelled monoclonal antibody in irradiated colon-cancer xenografts. This provides a proof of principle that irradiation can modify antibody accumulation, but whole-tumor uptake does not establish the uniform cellular exposure or productive delivery of a conjugated payload.

Brown et al. observed acute vascular changes after a single 20-Gy exposure in an intracranial tumor model; 6 Gy did not significantly change the measured parameters [5]. Sabatasso et al. studied the permeability after spatially fractionated microbeam irradiation using non-ADC probes [25]. Neither result establishes improved intact-ADC delivery after conventional clinical fractionation.

A key unresolved distinction is that between tumor entry and intratumoral penetration. Increased antibody accumulation near perfused vessels may raise whole-tumor uptake while leaving more distant antigen-positive compartments inadequately exposed. Bulk measurements of the tumor-associated antibody should therefore be complemented by spatial localization and, where feasible, measurements of released payload. These approaches are needed to determine whether irradiation improves pharmacologically meaningful drug exposure rather than simply increasing the total tumor-associated signal.

Antibody-based positron emission tomography (immunoPET) can reveal spatial accessibility, but is an indirect measure of ADC pharmacology. Williams et al. associated antibody uptake with ADC activity in some prostate-cancer models, with exceptions despite the substantial uptake [26]. The ZEPHIR study combined HER2 imaging with early metabolic assessment to identify a low likelihood of the T-DM1 benefit [27]. Both studies lacked RT and do not validate imaging as a measure of radiation-induced payload release.

Serial imaging in ADC–RT studies should therefore be used to test a predefined delivery hypothesis. Increased tracer uptake after irradiation may reflect changes in perfusion, vascular permeability, target availability, or nonspecific retention, and imaging alone cannot distinguish among these mechanisms. Pairing imaging with tissue pharmacodynamic measurements and, where feasible, a comparison with non-irradiated lesions would strengthen the mechanistic interpretation. The current evidence supports investigating how RT influences ADC delivery and sequencing, but it does not establish a universal advantage for administering RT before the ADC.

## 6. Linker Activation and Payload–Radiation Interactions

### 6.1. Radiation-Triggered Release Is a Distinct Therapeutic Strategy

Quintana et al. measured X-ray-triggered doxorubicin or MMAE release from engineered radiation-cleavable conjugates, with oxygen-dependent activation [18]. The platforms included non-antibody carriers and preirradiated-conjugate experiments. They establish chemical feasibility, not the in vivo selective activation of marketed ADCs by external-beam RT.

The translation of this strategy requires more than spatially confined radiation delivery. The payload released within an irradiated region may subsequently diffuse beyond the treatment field, while the activation efficiency may vary across heterogeneous tissue environments. Clinically useful constructs must therefore combine adequate circulation stability with efficient intratumoral activation and limited release in exposed normal tissues.

Radiopharmaceutical-triggered prodrug systems provide additional evidence that radiation-mediated drug activation is technically feasible [28]. However, these platforms are mechanistically adjacent to, rather than interchangeable with, conventional ADC–RT combinations. Their development requires the coordinated optimization of drug exposure and radiation delivery because both contribute to the effective pharmacological dose.

### 6.2. Anti-Tubulin Payloads: Radiosensitization Beyond Monotherapy Potency

Microtubule-targeting drugs can affect the radiation response outside mitosis. Markowitz et al. demonstrated an interphase contribution using free agents [29]. Application to an intact ADC requires evidence that the relevant intracellular payload concentration overlaps radiation injury and repair.

Hingorani et al. combined auristatin conjugates with fractionated RT in immunocompetent tumor models and used CD8 depletion and rechallenge to test immune contributions [7]. The study included antibody and activatable peptide carriers. Their dose units, activation requirements, and individual experimental arms must be distinguished (Supplementary Table S1).

Lesperance et al. provide both payload-level and intact-conjugate evidence [19]. In free-payload experiments, DXd more effectively prevented tumor establishment after the implantation of treated cells, whereas low-exposure MMAE showed stronger radiation interaction in the tested conditions. That implantation assay addresses immune protection against tumor establishment, not the treatment of an existing lesion. In established B16 tumors, human epidermal growth factor receptor 3 (HER3)-directed CDX3379-MMAE and CDX3379-DXd were compared at 2.5 nmol per ADC dose, twice 48 h apart, with 5 Gy × 3 beginning the day after the first dose. The MMAE ADC improved control relative to RT alone; the DXd ADC did not significantly improve that comparison under the tested conditions. Although the target and drug-to-antibody ratio were matched, the linker chemistry differed. Separate peptide-conjugate and immune-profiling arms should not be attributed to the ADC arm.

Combination studies should therefore distinguish additive cytotoxicity from the genuine modification of radiation response. A greater tumor-growth inhibition than either monotherapy alone is insufficient to demonstrate synergy. Dose–response analysis, appropriate interaction models, and durable-control endpoints are required to determine whether a payload meaningfully improves the radiation therapeutic index.

### 6.3. Topoisomerase-I Payloads: Mechanistic Plausibility Does Not Resolve Sequencing

Topoisomerase-I inhibitor ADCs deliver DNA-damaging payloads, but this mechanism alone does not establish radiosensitization or define an optimal treatment sequence. Abelman et al. [30] identified resistance to SN-38 and DXd associated with altered topoisomerase I (TOP1) function, addressing payload susceptibility rather than radiation timing. For intact ADCs, sequencing must therefore account for target engagement, linker processing, and the timing of released-payload exposure rather than being inferred from payload class alone.

The Lesperance experiments show why monotherapy potency cannot rank radiation combinations [19]. Their conclusions are conditional on the dose, exposure, carrier, linker, model, and endpoint; they do not establish that DXd-containing ADCs lack clinical radiosensitizing activity.

Acquired resistance further complicates interpretation. Abelman et al. [30] identified *TOP1* mutations associated with the resistance to topoisomerase-I ADCs and demonstrated functional cross-resistance, while Rampa et al. [31] provided evidence that payload diversification can restore activity in selected resistant models. Neither study demonstrates that RT reverses resistance to a compromised payload mechanism.

Clinical studies should distinguish ADC-naive disease from progression during prior ADC therapy and document previous payload classes. Local control after RT during ADC resistance can represent radiation control of a resistant lesion without the restoration of ADC sensitivity.

## 7. Immune Conversion: Functional Evidence and Competing Effects

### 7.1. Immunogenic Cell Death Markers and Protective Immunity

Calreticulin exposure, extracellular adenosine triphosphate (ATP), and high-mobility group box 1 (HMGB1) release are markers associated with immunogenic cell death (ICD). Consensus guidance requires functional evidence to establish a protective immune response; biochemical markers alone are insufficient [32].

Without RT, Bauzon et al. demonstrated ICD-associated features in vitro after maytansine-bearing ADCs [33]. Brentuximab vedotin and belantamab mafodotin studies provide functional immune evidence in their respective experimental systems [34,35]. These results support conjugate-specific hypotheses rather than a uniform immune property of a payload class.

Iwata et al. reported immune changes and enhanced programmed cell death protein 1 (PD-1) blockade activity with T-DXd [36]. Tsao et al. linked extracellular T-DXd processing with Toll-like receptor 4 (TLR4)/stimulator of interferon gene (STING)-associated myeloid activation [16]. Neither study included RT, so the incremental radiation contribution remains untested.

For ADC–RT combinations, the key unresolved question is whether irradiation adds a functional immune contribution beyond that generated by either treatment alone. CD8-depletion experiments, tumor rechallenge, and the assessment of non-irradiated lesions can strengthen causal inference. Increased immune-cell infiltration or inflammatory signaling alone is insufficient to demonstrate systemic antitumor immunity.

### 7.2. Convergent Innate Signaling May Not Produce Additive Benefit

Wu et al. reported cyclic GMP–AMP synthase (cGAS)–STING activation and enhanced immunotherapy activity with disitamab vedotin in HER2-positive colon-cancer models without RT [37]. Combining two innate stimuli could be beneficial, redundant, or harmful; pathway convergence alone does not resolve the outcome.

Radiation fractionation adds further complexity. Vanpouille-Box et al. [8] demonstrated that sufficiently high single radiation doses induced TREX1, reduced cytosolic DNA accumulation, and attenuated interferon signaling in the models studied. The reported threshold varied between approximately 12 and 18 Gy and should not be interpreted as a universal clinical ceiling or directly translated into ADC–RT scheduling recommendations.

Testing an innate-immune mechanism requires the perturbation of the relevant pathway or cell population and a functional endpoint. Transcript or cytokine changes can guide sampling, but should remain supportive measurements.

### 7.3. Immune Activation Can Coexist with Immune Depletion

Xing et al. observed C-C motif chemokine ligand 8 (CCL8)-associated M2-like macrophage signals after low-dose RT and lower signals with combination treatment [17]. Reduced staining cannot distinguish direct killing from altered recruitment or tissue composition. The antibody’s lack of mouse ICAM1 binding further limits a direct murine-macrophage targeting interpretation.

Clinical evidence similarly challenges the assumption that RT consistently enhances useful antitumor immunity. Zenga et al. [9] identified the preferential depletion of tumor-antigen-specific lymphocytes after preoperative hypofractionated RT in head and neck cancer. This finding supports the assessment of T-cell identity and function rather than reliance on total lymphocyte infiltration as a surrogate for effective immunity. Its relevance across tumor types and fractionation regimens remains uncertain.

Radiation-related lymphopenia represents an additional systemic constraint. Reviews have linked lymphocyte depletion to treatment volume, dose distribution, and the exposure of lymphocyte-rich tissues, while also documenting substantial heterogeneity [38,39]. Associations with inferior outcomes do not, however, demonstrate that lymphocyte-sparing RT improves survival or enhances ADC–RT efficacy.

Prospective studies should therefore integrate longitudinal lymphocyte measurements and relevant dosimetry with tumor immune profiling. This would allow investigators to determine whether local immune activation is accompanied by the preservation of the systemic immune capacity required for sustained antitumor effects.

### 7.4. Immune-Stimulating Conjugates Require Separate Combination Hypotheses

Conjugates delivering innate immune agonists represent a distinct therapeutic strategy and require their own rationale for combination with RT. Bukhalid et al. [40] demonstrated antitumor immune activity with the HER2-directed STING-agonist conjugate XMT-2056, whereas Appleman et al. [41] investigated the C-C motif chemokine receptor 2 (CCR2)-directed delivery of TAK-500 to intratumoral myeloid cells. Because these platforms act in different cellular compartments, they should not be treated as mechanistically interchangeable.

RT could theoretically provide tumor-derived antigenic material while the conjugate enhances innate immune activation. That division of function remains a hypothesis, however, rather than an established consequence of the cited studies. Development should determine whether irradiation adds functional antigen presentation and tumor control beyond the conjugate alone and whether targeted delivery sufficiently limits systemic inflammatory exposure.

## 8. A Four-Window Framework for Sequencing

**Figure 2:**
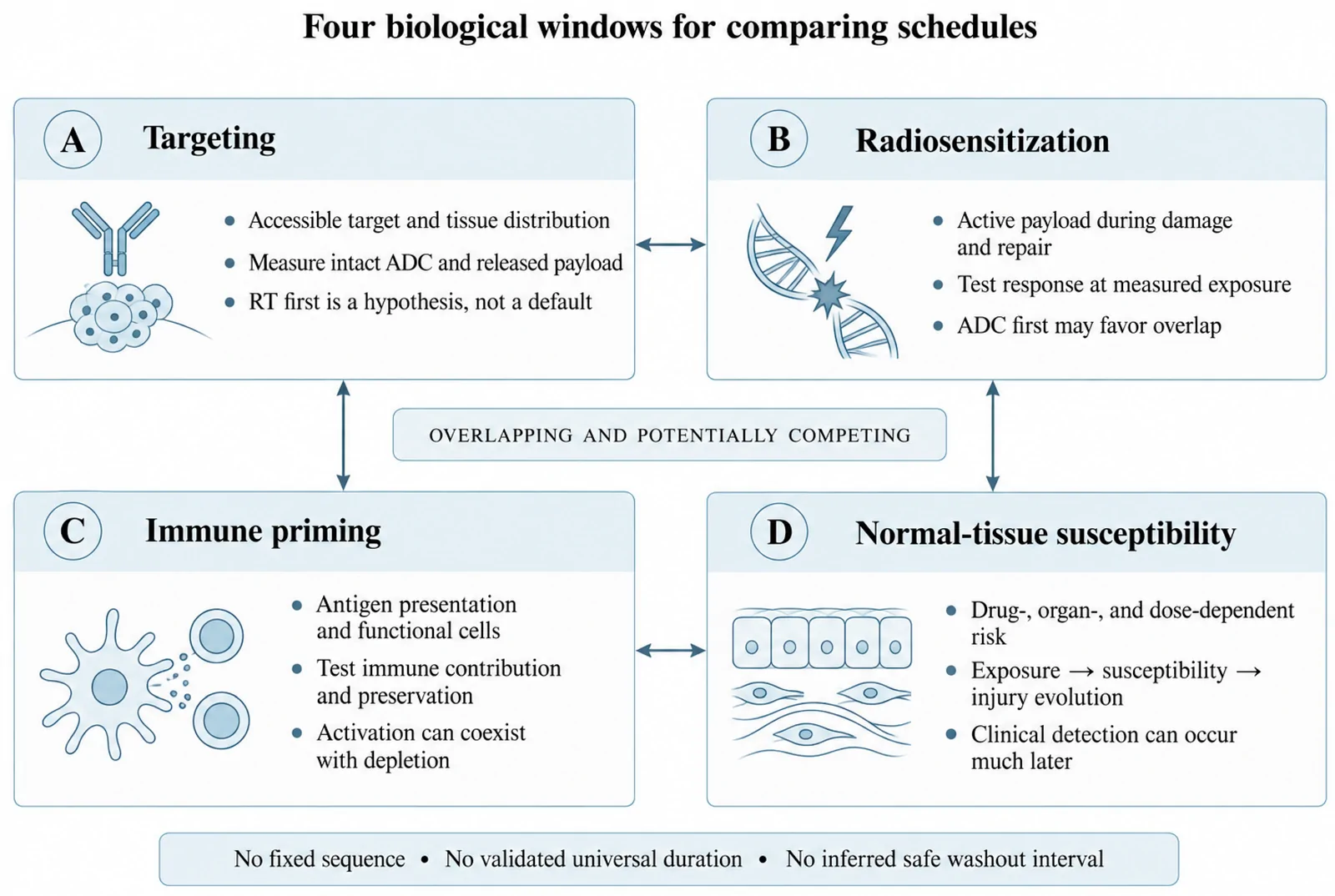
Four overlapping and potentially competing biological windows. Targeting, radiosensitization, immune priming, and normal-tissue susceptibility can coexist and influence one another; their positions do not imply a fixed sequence or validated duration. Exposure overlap, biological susceptibility, injury evolution, and clinical detection are distinct. A late toxicity event establishes the need for surveillance, not the timing of the initiating interaction. ADC, antibody–drug conjugate; RT, radiotherapy.

The four windows describe processes that can favor different schedules. RT before an ADC might increase target accessibility, whereas an ADC before RT might establish active payload exposure during damage and repair. The same exposure overlap could increase normal-tissue injury or deplete useful immune cells. The framework makes these tradeoffs explicit (Figure 2).

Its intended output is a testable schedule comparison, not a recommendation based on elapsed time alone. Investigators should name the proposed mechanism, select a competing schedule, collect the measurements needed to distinguish them, and evaluate tumor control alongside organ-specific harm. Onset and duration remain unknown for most clinical combinations.

### 8.1. Targeting Window

When RT is intended to enhance ADC delivery, the relevant window is the interval during which irradiation increases productive exposure in a viable tumor. Increased antigen expression, vascular entry, and internalization may all contribute, but they are not interchangeable endpoints. Demonstrating one does not establish that sufficient active payload reaches residual tumor cells.

TIP1- and ICAM1-directed experiments justify testing radiation-responsive targets, but provide no transferable interval for HER2-, TROP2-, or nectin-4-directed ADCs [6,17]. Serial sampling must connect the accessible target with the distribution and processing of the actual conjugate. Unconjugated-antibody fluorescence remains a surrogate.

A priming dose should be compared with an alternative sequence at the same intended ADC and RT doses. Lower-dose priming is a research strategy; it cannot be preferred clinically without evidence of improved benefit and harm.

### 8.2. Radiosensitization Window

For payload-mediated radiosensitization, treatment timing should be linked to the presence of a biologically active payload during radiation-induced injury and repair. The interval between ADC infusion and irradiation is clinically observable but remains an imperfect surrogate for intracellular drug exposure. The persistence of the circulating antibody or intact conjugate does not demonstrate sustained pharmacological activity within tumor cells.

Free-payload and intact-conjugate experiments address different parts of the radiosensitization question [19]. An infusion date is an exposure marker, not the time at which the intracellular payload necessarily reaches its maximum effect.

Studies should therefore report exact ADC administration dates, individual radiation fractions, dose modifications, interruptions, and prior ADC exposure. Where feasible, pharmacokinetic and pharmacodynamic measurements should distinguish the intact conjugate from the released payload. Retrospective definitions of “concurrent” treatment remain useful for classifying exposure, but cannot identify the biological interval responsible for efficacy or toxicity.

### 8.3. Immune Priming Window

The immune priming window concerns tumor damage, antigen presentation, and the availability of functional responding cells. ICD-associated markers are useful early readouts, while immune-dependent tumor control requires functional confirmation [32].

The preclinical evidence of CD8-dependent tumor control supports the investigation of immune contributions to ADC–RT combinations [7]. Conversely, the depletion of tumor-reactive lymphocytes following irradiation demonstrates that antigen release can coexist with the loss of the cells required to exploit it [9]. These competing effects preclude the inference of an optimal sequence from inflammatory biomarkers alone.

For an immune substudy, prioritize the cell population or pathway that mediates the hypothesis. Tumor-reactivity assays and functional perturbation provide stronger evidence than cytokines or clonality alone. Repeated tissue or draining-node sampling is optional and should be justified by the specific question.

### 8.4. Normal Tissue Injury Window

Four intervals must be distinguished: drug–RT exposure overlap; biological tissue susceptibility; subsequent injury evolution; and eventual clinical detection. They need not coincide. COMBART detected a grade 3 radionecrosis event at 16 months, and a bladder series reported hemorrhagic cystitis at 20 and 22 months [42,43]. These observations justify prolonged surveillance but do not identify when the initiating interaction occurred.

The risk must remain specific to the conjugate and irradiated organ. The intracranial experience with T-DM1 cannot be directly extrapolated to T-DXd, and reassuring findings from limited palliative fields cannot establish safety for large thoracic or pelvic volumes, reirradiation, or other high-risk settings. Previous RT, target volume, cumulative organ exposure, and pre-existing toxicity should therefore be evaluated alongside treatment timing.

A susceptibility window cannot be estimated from plasma clearance or toxicity latency alone. The relevant schedule comparison must preserve the proposed tumor interaction while testing whether clinically meaningful organ injury is reduced. No universal washout interval follows from this framework.

### 8.5. Worked Example of a Targeting Hypothesis

The ICAM1 study illustrates an experiment-driven schedule hypothesis, not a clinical prescription [17]. After 2 Gy, ICAM1 rose by 12 h and approached a plateau at 48 h in the tested cells; persistence beyond a week was observed in HCT116 cells. Induction was model-dependent: SUM190 cells showed a stronger response to 2 Gy × 3 than to a single fraction. A separate uptake experiment administered the fluorescent unconjugated antibody 24 h after 2 Gy. That interval must not be assumed to be the validated optimum for the intact ADC efficacy experiment.

A prospective test could compare RT followed by the ICAM1–DXd ADC with ADC followed by RT, using the same total doses, plus ADC-alone, RT-alone, and vehicle controls. Baseline and 12-, 24-, and 48-h samples would test the reported induction time course; later sampling would assess persistence. Those sampling times are proposed design choices. Measuring surface ICAM1, spatial intact-ADC distribution, and released DXd would test the delivery pathway; tumor control at a comparable exposure would support a response-mediated alternative. Relevant cross-reactive normal-tissue models are needed before translating the schedule. CD8 dependence cannot be inferred from the original nude-mouse xenografts.

### 8.6. Applying the Windows to Clinical ADCs

For T-DM1 with brain SRS, the dominant constraint is the observed radionecrosis association; a timing study should stratify the prior RT, lesion volume, dose/fractionation, and baseline neurologic status, with symptomatic radionecrosis and durable local control as co-relevant outcomes [11]. Aquaporin-4 (AQP4) is a candidate mechanistic measure, not a validated patient-selection biomarker. These data do not specify a safe holding interval.

For T-DXd, separate a tumor radiosensitization hypothesis from the pulmonary injury question. Record the exact infusion/fraction dates, baseline lung disease, and lung dose–volume exposure; compare local control and treatment continuity with adjudicated interstitial lung disease (ILD)/pneumonitis and delayed central nervous system (CNS) injury [10,42,44]. Serial DXd exposure or DNA-damage measurements are optional mechanistic tools, not established scheduling tests.

For SG, SN-38 exposure provides a plausible radiosensitization mechanism, while marrow and bowel exposure may constrain the overlap. Record the blood counts, gastrointestinal symptoms, pelvic/marrow dosimetry, dose interruptions, and previous payload treatment [45,46]. A schedule comparison should assess sustained local or symptom control and systemic-treatment delivery alongside hematologic and gastrointestinal toxicity. TROP2 or TOP1 status alone does not define a clinical timing window.

## 9. Evidence Map: Separating Demonstration from Inference

The ADC–RT evidence base contains several fundamentally different forms of support, including direct mechanistic experiments, pharmacodynamic observations, toxicity associations, and treatment-feasibility cohorts. These study types answer different questions. A controlled preclinical experiment may establish a mechanism within a defined model without demonstrating a clinical benefit, whereas a clinical cohort may show that treatment can be delivered without establishing why it was effective or whether an alternative schedule would have been safer.

Table 1 separates direct ADC–RT experiments, ADC experiments without RT, indirect component studies, and clinical associations. Supplementary Table S1 adds the actual models, agents, radiation schedules, controls, and endpoints. These categories describe the evidentiary scope rather than a single ladder: a clinical toxicity association and a mechanistic mouse experiment answer different questions.

A further distinction is required between demonstrating a treatment effect and attributing that effect to an interaction. The local response after combined therapy may reflect RT activity, ADC activity, or a biological interaction between them. Likewise, an adverse event occurring during combination treatment does not independently identify its causal component. Comparative schedules, appropriate controls, and mechanism-specific pharmacodynamic measurements are required to connect the observed outcomes to the proposed interaction.

## 10. Payload-Informed Combination Design

Payload identity is an important determinant of ADC–RT behavior, but it cannot be considered independently of the intact conjugate. Antibody specificity, linker stability, the drug-to-antibody ratio, internalization, extracellular processing, and pharmacokinetics collectively determine where and when the active payload becomes available. ADCs carrying related payloads may therefore differ substantially in their interactions with RT, particularly in normal tissues.

The comparative findings of Lesperance et al. [19] support the direct evaluation of radiation interactions rather than ranking combinations according to ADC monotherapy potency. Likewise, the differing clinical profiles of T-DM1 and T-DXd cannot be attributed solely to their payloads because these agents also differ in linker chemistry, drug loading, bystander properties, and pharmacological behavior.

Table 2 translates these considerations into testable development questions. It separates the observed or plausible liabilities from proposed scheduling strategies. At present, neither a class-wide treatment interval nor a preferred radiation-fractionation strategy can be derived from payload mechanism alone.

Prior treatment history is another determinant of combination design. Persistent antigen expression after progression does not establish continued susceptibility to the same payload mechanism. TOP1-associated resistance provides a clear example of a downstream limitation that RT has not been shown to reverse [30]. Studies should therefore distinguish the treatment of ADC-sensitive disease from local irradiation performed during systemic ADC resistance. In the latter setting, RT may control resistant lesions while allowing a continued systemic benefit elsewhere without demonstrating the restoration of ADC sensitivity.

## 11. Clinical Evidence: What Feasibility Studies Can and Cannot Establish

Clinical evidence should be interpreted according to treatment intent, irradiated organ, and the comparison actually performed. Palliative RT delivered during systemic ADC therapy, the integration of an ADC into curative locoregional treatment, and the stereotactic irradiation of brain metastases represent fundamentally different clinical scenarios. Their outcomes should not be combined into a general statement that concurrent ADC–RT treatment is “safe”.

Most dedicated combination reports are observational. Selected studies are discussed below by ADC and setting; Supplementary Table S2 adds radiation exposure, concurrency definitions, previous RT, follow-up, endpoint units, and verification limits. Randomized systemic-treatment trials provide context but do not test the effect of RT timing.

### 11.1. Trastuzumab Deruxtecan: Expanding Evidence with Persistent Organ-Specific Uncertainty

TENDANCE included 54 patients receiving T-DXd and RT, with no RT-related T-DXd discontinuation during a median nine-month follow-up [10]. The median interval from the last infusion to RT was 10 days (range, 1–21), and from RT completion to the next infusion was 11 days (range, 3–25). The varied palliative and ablative regimens demonstrate treatment delivery under those conditions; they do not define an optimal interval or exclude delayed injury.

COMBART prospectively followed 40 patients with 98 irradiated lesions for a median 11 months (range, 6–31) [42]. The 21 brain and 34 thoracic lesions are lesion counts, not organ-specific patient denominators; only five thoracic lesions were lung lesions. Four patients experienced late toxicity, with three pneumonitis and three radionecrosis events across the cohort. These six events must not be reported as six affected patients. A grade 3 radionecrosis event was detected at 16 months. Among 22 patients treated for oligoprogression, the median time to systemic-treatment change was 19.1 months (95% confidence interval [CI], 12.7–25.56); the uncontrolled design cannot establish that RT caused this treatment continuity.

Visani et al. compared 67 patients receiving concomitant RT with 80 receiving T-DXd alone [47]. Grade ≥3 adverse events occurred in 8/67 (11.9%) versus 16/80 (20.0%; *p* = 0.30), with a median follow-up of nine months overall. RT recipients were younger and more often had brain metastases; no lung metastases were irradiated. The nonsignificant difference does not establish equivalent safety, particularly for thoracic RT.

DESTINY-Breast05 randomized 1635 patients with high-risk residual HER2-positive early breast cancer to T-DXd or T-DM1; adjudicated drug-related ILD occurred in 9.6% and 1.6%, respectively [44]. A subsequent conference abstract addresses pulmonary safety [52]. It is a secondary analysis of the same trial, not an independent cohort or a randomized test of RT timing. Drug-related ILD and radiation pneumonitis remain distinct endpoints even when their clinical features overlap.

Khatri et al. reported 215 lesions across 48 SRS courses in 34 T-DXd-treated patients [53]. At 24 months, the cumulative symptomatic radionecrosis incidence was 2.1% per lesion and 11% per patient; these measure different risks. The reported local control was 97% at 12 months and 88% at 24 months. Without an appropriate comparator, high local control does not establish ADC-mediated radiosensitization.

Chun et al. studied 113 patients, including 29 receiving T-DXd, and 461 SRS treatments [54]. No radionecrosis was observed in the T-DXd group, but the mean treatment-level follow-up was shorter for the T-DXd-exposed than comparison treatments (15.5 versus 28.1 months). The study also used endpoint-dependent exposure classifications. Selection, prior RT, fractionation, repeated treatments, competing death, and unequal observation time limit causal comparisons; the findings cannot establish zero risk or a protective effect.

Pulmonary injury requires a separate attribution process. With T-DXd, evaluate symptom onset relative to each infusion and RT course, baseline imaging, infection and other competing causes, and the distribution of new opacities against the dose map. Predominantly in-field changes support a radiation contribution, whereas out-of-field disease can support a drug-related or other cause; neither pattern is definitive. Record drug-related ILD, radiation pneumonitis, mixed injury, and indeterminate cases separately through clinical/radiologic adjudication, with grade, treatment, recovery, and drug rechallenge [42,44,52]. Such attribution should inform the analyses without concealing the total pulmonary event burden.

### 11.2. T-DM1 and Intracranial Radionecrosis

Stumpf et al. reported clinically significant radionecrosis in 9/23 T-DM1-exposed patients (39.1%) and 1/22 unexposed patients (4.5%) in a selected 45-patient cohort [11]. The reported odds ratio was 13.5 (95% CI, 1.5–118.7), indicating substantial uncertainty. Exposure included nonconcurrent treatment histories; these proportions do not isolate the risk from simultaneous administration.

Koide et al. studied 168 patients, including 19 with ADC exposure within four weeks before or after brain RT [49]. With a median follow-up of 31 months, the two-year symptomatic radionecrosis incidence was 27% versus 7%; 33% of the overall cohort had previous brain irradiation. The competing-risk analysis addresses delayed injury more directly than crude event proportions, but the small mixed-agent exposed group and prior-treatment differences still limit causal interpretation.

Salvestrini et al. estimated a pooled grade ≥3 radionecrosis incidence of 17% in the included T-DM1 literature [51]. Zare et al. synthesized seven studies and estimated symptomatic radionecrosis in 20% of ADC recipients (95% CI, 12–32%) [55]. These are different endpoints. The syntheses reuse published cohorts and must not be added to them as independent patients. The variation in ADC, concurrency, fractionation, lesion risk, and follow-up limits the interpretation of pooled estimates as a drug-specific absolute risk.

Shen et al. provide an additional small observational brain-RT series involving T-DM1 and T-DXd [56]. Such mixed-agent cohorts are informative only when events and denominators can be assigned to the specific drug, timing category, and irradiation history.

A plausible tissue mechanism accompanies the epidemiologic signal. In human astrocyte experiments, Stumpf et al. associated T-DM1 plus irradiation with AQP4 dysregulation and astrocytic swelling; limited clinical specimens showed reactive astrocytic changes [11]. This supports a candidate injury pathway, not a universal mechanism of human radionecrosis or a validated washout interval. Clinical analyses still require lesion volume, spatial dosimetry, prior RT, survival, and the adjudication of tumor progression versus radiation injury.

Extracranial toxicity requires a separate interpretation. Dastgheyb et al. [50] reported grade 3 acute skin toxicity in 3 of 35 patients receiving concurrent T-DM1 with breast or chest-wall RT. The grade ≥2 count differs between the abstract and main results and is therefore not reproduced here. These findings justify the attention to locoregional toxicity but do not imply that the intracranial risk profile applies to breast irradiation.

### 11.3. Sacituzumab Govitecan and Enfortumab Vedotin

SG evidence includes the palliative cohorts of Krug et al. and Loap et al. [45,46]. These small selected series support further evaluation, but cannot establish a schedule for extensive marrow, bowel, or thoracic irradiation. Baseline cytopenias, diarrhea, irradiated volume, other systemic treatments, and dose interruption are important modifiers.

ATTENTION included 55 patients and 63 irradiated lesions, with 37 lesions classified as concurrent using RT within four days before or after SG [48]. No grade ≥3 toxicity was reported with concomitant SG, but the median follow-up was 7.9 months (range, 1.7–35.4). This selected retrospective experience does not validate a four-day safety interval or exclude delayed organ injury.

Seyedin et al. reported early enfortumab vedotin (EV)–RT feasibility [57]. Lock et al. subsequently included 103 EV- or SG-treated patients and 166 RT courses; 77 concurrent courses occurred in 60 patients [58]. Concurrent treatment meant RT initiation within four weeks before or after ADC treatment. Ten of these 60 patients stopped the ADC for grade 3–5 toxicity. Investigators considered only one case potentially influenced by RT: neutropenia and fatal pneumonia after SG with craniospinal irradiation. This attribution should be reported alongside, rather than replace, the full high-grade toxicity burden.

Prospective EV studies address different bladder-cancer questions. STAR-EV (NCT06394570) examines neoadjuvant EV and stereotactic RT before cystectomy; CONSOLIDATE (NCT06434350) examines concurrent EV–RT for locally advanced disease; and EV-PRIME (NCT06470282) examines EV, pembrolizumab, and bladder-preserving RT [59,60,61]. These protocols are evidence of planned investigation, not demonstrated safety or efficacy.

### 11.4. Bladder Preservation and Lymphoma: Early Signals Requiring Distinct Endpoints

The transition from palliative treatment to bladder preservation raises a substantially higher evidentiary threshold. Zhang et al. [62] reported six patients in the first stage of a phase II study combining disitamab vedotin, toripalimab, and RT. Circulating tumor DNA (ctDNA) and urinary tumor-DNA measurements provide an exploratory approach to response monitoring, but the molecular response cannot substitute for durable local control, preserved bladder function, and the avoidance of salvage cystectomy.

Chen et al. reported ten patients receiving disitamab vedotin, toripalimab, and bladder/pelvic RT [43]. Six had cT1 disease, one cT2, two cT3, and one cT4a; the outcomes therefore cannot be compared directly with a muscle-invasive-only cohort. Nine completed RT and one had grade 3 leukopenia. Nine developed grade 1–2 radiation cystitis, and two had hemorrhagic cystitis at 20 and 22 months. The median follow-up was 17.2 months (range, 6–31). Nine retained their bladder, but this descriptive endpoint does not establish preserved function or isolate the ADC contribution.

Melancon et al. describe polatuzumab vedotin with RT in 21 patients with heavily pretreated aggressive B-cell lymphoma [63]. The report is relevant to baseline marrow vulnerability and combined-treatment tolerability, but a detailed quantitative interpretation requires the full study methods and results (Supplementary Table S2).

The mycosis fungoides report by Oymanns et al. [64] provides another hypothesis-generating observation involving an ADC and radiation-based treatment. Durable remission after a multimodal regimen cannot independently establish radiosensitization or attribute a benefit to any individual component.

Prospective protocols are beginning to incorporate explicit sequencing. PRaG3.0 combines disitamab vedotin with hypofractionated RT and multiple immune-modulating therapies [65]. Such designs create opportunities for pharmacodynamic investigation but also substantial attribution challenges. Favorable outcomes in a single-arm multicomponent regimen would not establish either an optimal sequence or a specific ADC–RT interaction. Table 3 summarizes the selected clinical evidence and its principal interpretive limitations.

### 11.5. What the Clinical Evidence Currently Supports

Clinical experience supports selected use and prospective investigation, with the strongest uncertainty concerning incremental benefit and delayed organ injury. A palliative bone field, SRS to a previously irradiated brain, and definitive pelvic RT expose different tissues and cannot supply interchangeable safety estimates.

The apparent differences between reports may reflect treatment selection, prior irradiation, lesion size, dose and fractionation, concurrent-treatment definition, co-medications, or follow-up rather than a true difference between ADCs. A shorter observation and earlier death reduce opportunities to detect late events. Analyses should account for competing death and multiple lesions or courses per patient, report confidence intervals, and avoid interpreting a nonsignificant comparison as an equivalence.

The clinically useful endpoint is a balance: durable local control, symptom relief, organ preservation, and systemic-treatment continuity weighed against acute and delayed harm. These outcomes require defined comparators and cannot be replaced by the response rate alone.

## 12. Resistance as a Dynamic and Spatial Problem

ADC resistance should be interpreted according to the biological level at which activity fails. Reduced target availability, inadequate tissue penetration, impaired intracellular processing, and resistance to the released payload represent distinct mechanisms. An increasing target expression is unlikely to restore sensitivity when the dominant limitation lies downstream of binding. In neuroblastoma models, Buongervino et al. [66] demonstrated that target density, antibody internalization, and payload susceptibility jointly influenced ADC activity, illustrating the need to evaluate several determinants of the response.

This distinction becomes particularly important when RT is proposed as a resistance-modifying intervention. Improved control of an irradiated lesion may reflect direct radiation cytotoxicity, increased ADC delivery, enhanced payload activity, or a combination of these effects. Only the latter two would constitute evidence that RT modified ADC sensitivity. Conversely, progression after combined treatment does not demonstrate the radiation-induced selection of resistant clones. Such a conclusion requires longitudinal evidence linking a treatment-associated molecular alteration to a functional resistance phenotype, ideally with a comparison against ADC treatment alone.

Zhao et al. [67] provide an instructive example of the coordinated modulation of target expression and payload susceptibility. In preclinical breast-cancer models, decitabine increased both the TROP2 and schlafen family member 11 (SLFN11) expression and enhanced SG activity. Because this was an epigenetic rather than radiation-based intervention, its relevance to ADC–RT treatment is conceptual: an increasing target abundance may be insufficient if susceptibility to the delivered payload is not preserved. Whether RT can induce a comparable combination of target and payload-sensitivity changes requires a direct assessment.

Prior ADC exposure should therefore be treated as a biological variable. Abelman et al. [30] identified acquired *TOP1* alterations in patients progressing on topoisomerase-I ADCs and demonstrated functional cross-resistance. The retention of the target recognized by a subsequent ADC cannot therefore guarantee sensitivity when the payload mechanism is shared. These findings do not, however, establish *TOP1* mutation testing as a validated exclusion criterion for subsequent therapy.

Rampa et al. [31] further showed that switching to ADCs with mechanistically distinct payloads could restore activity in resistant preclinical breast-cancer models. These data support the prospective evaluation of payload diversification but do not establish a universal sequencing strategy. For ADC–RT studies, the relevant question is whether irradiation modifies the specific resistance mechanism operating within the treated lesion. Progression sampling should therefore distinguish among target loss, impaired delivery, defective processing, and payload resistance where feasible, while recognizing that different lesions within the same patient may harbor different mechanisms.

## 13. Biomarkers for a Radiation-Conditioned ADC State

The biomarker assessment should be proportionate to the claim. Every clinical study needs the core exposure, outcome, and toxicity dataset described in Section 14.4. Serial biopsies, immunoPET, tissue drug assays, and tumor-reactive T-cell studies belong in selected mechanistic substudies rather than being mandatory for all participants.

Whole-body target imaging can complement tissue sampling by identifying inter-lesional heterogeneity. The ZEPHIR study illustrates the potential value of combining HER2-targeted imaging with an early response assessment during T-DM1 treatment [27]. However, immunoPET does not directly measure the intracellular payload release. An increased uptake after irradiation may reflect altered perfusion, vascular permeability, antigen availability, or tracer retention; interpreting it as improved therapeutic delivery therefore requires additional evidence.

For a delivery claim, a direct assay distinguishing the intact ADC and released payload is more informative than DNA-damage staining alone. For radiosensitization at a comparable exposure, the dose–response analysis and relevant pharmacodynamic timing are central. RT and cytotoxic payloads can produce overlapping damage markers, so attribution requires appropriate controls.

For an immune-mediated claim, functional immune dependence or tumor reactivity is more informative than inflammation alone [32]. In preclinical studies, depletion or pathway perturbation can test causality; clinical studies generally provide convergent evidence rather than an equivalent causal experiment.

Table 4 distinguishes the measurement most directly needed for each mechanistic claim from supportive options. Assay access should determine the substudy scope, not create unsupported biomarker thresholds for routine treatment selection.

## 14. Trial-Design Priorities

### 14.1. Select the Clinical Question and Mechanism Together

ADC–RT studies should begin with a clearly defined clinical problem and a testable biological explanation for why combining the treatments could improve that outcome. Improving local control, preserving an organ, controlling oligoprogression, and inducing systemic immune effects are distinct objectives that require different comparators, sampling strategies, and definitions of success.

A shared target antigen alone is insufficient justification for a basket design. Cross-histology enrollment is most informative when the proposed biological interaction, relevant exposure, and biomarker interpretation are sufficiently comparable across tumor types. Where treatment geometry or normal-tissue constraints dominate the clinical question, disease- or organ-specific cohorts are likely to provide more interpretable evidence.

**Figure 3:**
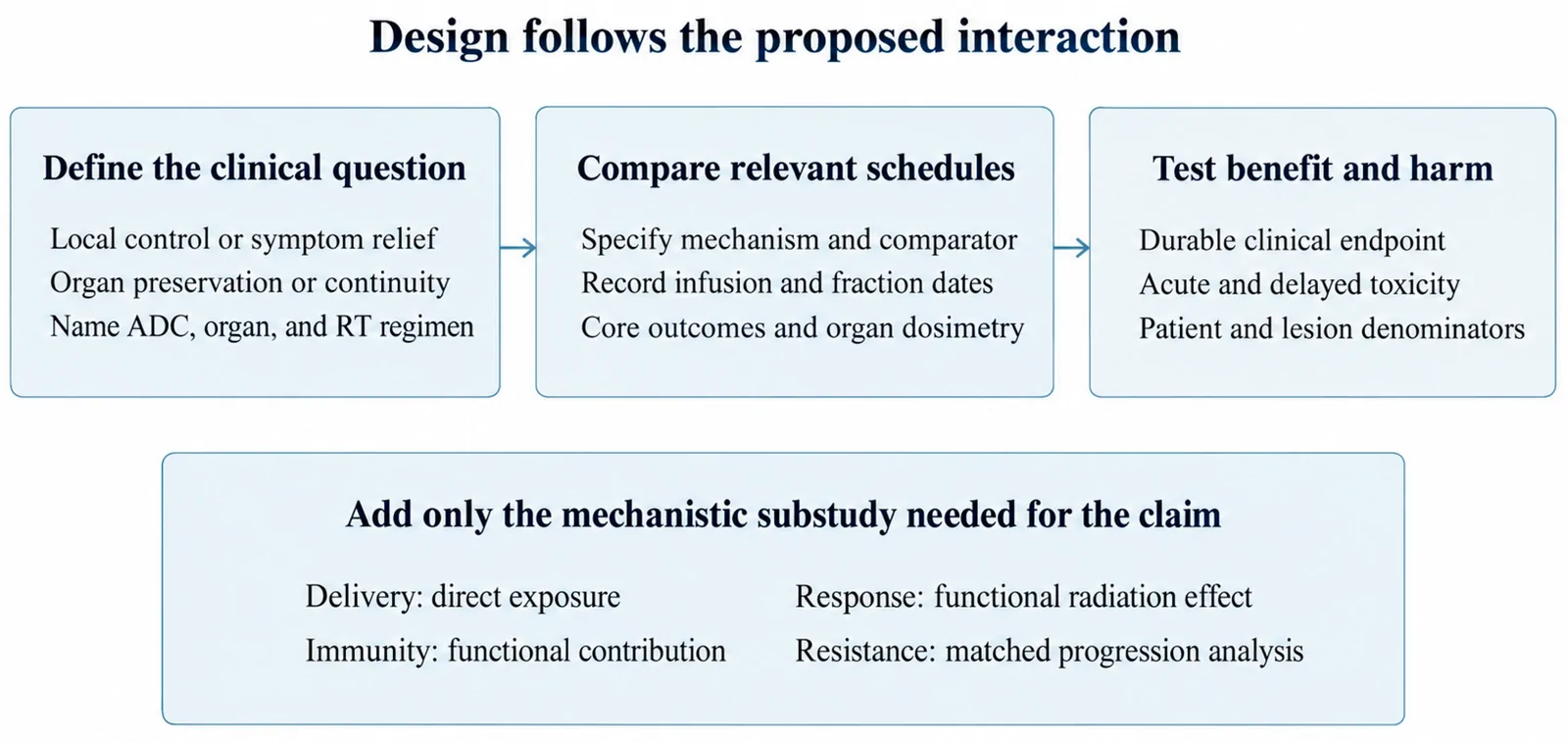
Design of studies comparing ADC–RT schedules. Define the clinical question, select a mechanism-informed comparator, and test durable benefit alongside acute and delayed harm. Exact treatment dates, organ dosimetry, and patient and lesion denominators form the core clinical dataset. Add the mechanistic substudy needed to assess delivery, radiation response, immunity, or resistance. This is a conceptual design framework, not a validated treatment-selection algorithm.

Early-phase studies can establish feasibility and identify candidate schedules, but uncontrolled response rates cannot separate combination activity from the independent effects of RT and the ADC. A randomized evaluation should follow once a schedule has adequate safety support and a credible mechanism. Figure 3 distinguishes the core clinical comparison from selected mechanistic substudies.

### 14.2. Dose, Fractionation and Timing Require Joint Consideration

Combination exposure is multidimensional. ADC dose and interval, radiation dose, fractionation, treated volume, treatment sequence, and previous exposure may all influence efficacy and toxicity. Attempting to vary every dimension simultaneously in a small early-phase study, however, would compromise both toxicity attribution and schedule interpretation.

Initial development should therefore focus on a limited number of biologically justified schedules. Where appropriate, established radiation parameters can be held constant while the ADC exposure or treatment interval is varied. Additional dimensions can then be explored once the emerging data provide sufficient justification. The aim should be to identify an informative and tolerable regimen rather than to maximize exposure to both treatments.

Observation periods must also match the toxicity under investigation. A conventional early dose-limiting toxicity window cannot exclude delayed radionecrosis, pneumonitis, or other late tissue injury. Decisions regarding expansion or advancement should therefore incorporate continuing organ-specific surveillance, treatment interruptions, recovery, and delayed adverse events rather than relying solely on the completion of the first treatment cycle.

### 14.3. Match Endpoints and Comparators to the Claim

Clinical benefit and mechanistic interaction are related but distinct questions. A randomized comparison of ADC plus RT versus ADC alone can estimate the benefit of adding radiation in a defined setting, but does not by itself demonstrate biological synergy. A radiosensitization claim requires evidence that the combination alters the radiation response beyond the prespecified expectation from the individual treatments. Factorial designs may help address this question where clinically and ethically feasible, but they are not appropriate in every setting.

For local-treatment objectives, studies should prespecify lesion-level control, the durability of the response, competing events, and the relationship between the lesion-level outcomes and patient-level benefit. Symptom control, the avoidance of local complications, and organ preservation may be more clinically meaningful than the radiographic response rate alone.

For immune-potentiation hypotheses, responses in non-irradiated lesions are informative but cannot independently establish an abscopal mechanism when the ADC or concomitant checkpoint inhibitor is systemically active. Stronger evidence requires appropriate comparators together with a spatial immune assessment and functional demonstration of tumor-specific immunity. Peripheral cytokine changes should remain supportive rather than definitive endpoints.

Claims of an improved therapeutic index require the evaluation of both efficacy and harm. Increased tumor exposure or tumor response should be assessed alongside normal-tissue exposure, radiation dosimetry, and clinically meaningful acute and delayed toxicity. A favorable tumor biomarker cannot compensate for an inadequately characterized organ-specific risk.

### 14.4. CNS Studies Require Dedicated Assessment

Studies combining ADCs with stereotactic radiosurgery should distinguish radiographic treatment change from symptomatic radionecrosis and define how progression, treatment effect, and mixed pathology will be adjudicated. Central imaging review, predefined radiological and clinical criteria, and pathology where available can improve consistency. Corticosteroid use, bevacizumab treatment, surgery, and neurological consequences should be reported alongside the toxicity grade.

Patient-level and lesion-level analyses answer different questions. As illustrated by the T-DXd/SRS cohort reported by Khatri et al. [53], lesion-level analyses estimate the event risk for individual treated metastases, whereas patient-level analyses describe the probability that a patient experiences clinically meaningful toxicity. Lesion-level models should account for the clustering of multiple lesions within individual patients, and both approaches require adequate follow-up and the consideration of death as a competing event.

The core dataset includes the lesion size and location, fractionation and dose–volume metrics, previous irradiation, and exact ADC dates. Table 5 separates these clinical reporting requirements from optional mechanistic assays.

### 14.5. Prospective Studies Address Different Therapeutic Questions

The emerging prospective landscape should not be interpreted as a single test of ADC–RT synergy. STAR-EV [59] evaluates neoadjuvant treatment before radical cystectomy, whereas EV-PRIME [61] investigates a bladder-preserving strategy. CONSOLIDATE [60] evaluates concurrent EV and RT in locally advanced bladder cancer. Although all involve EV, they address different clinical decisions and should be interpreted accordingly.

PRaG3.0 combines disitamab vedotin with radiation, checkpoint blockade, and cytokine support [65]. Such a design can evaluate the complete therapeutic regimen but cannot isolate the contribution of individual components. Similarly, the salivary-gland carcinoma study [68] introduces stereotactic RT after prespecified disease assessments during ongoing ADC treatment. Outcomes among patients who reach that treatment stage will necessarily be influenced by the preceding selection process.

Table 6 summarizes selected prospective studies. Trial registration documents the planned investigation; it should not be interpreted as evidence of efficacy, safety, successful recruitment, or completion.

## 15. Translational Research Priorities and Future Directions

Prioritize studies that compare plausible schedules within a defined ADC, organ, and clinical setting. Mechanistic sampling should establish whether a benefit is delivery-mediated or occurs at a comparable exposure, with a comparator that can distinguish between these explanations.

Mechanistic studies should also disentangle the contributions of the antibody, linker, payload, and pharmacokinetics. Matched experimental systems are preferable to comparisons between clinically distinct ADCs, in which several variables change simultaneously. Immunocompetent models are particularly important when immune potentiation is proposed, while radiation-responsive conjugates require a separate assessment of activation efficiency, spatial selectivity, and normal-tissue exposure.

Prospective studies need exact infusion and RT dates, relevant organ dosimetry, baseline vulnerability, and a follow-up appropriate to the injury under study. Organ-specific analyses should include the number of exposed patients, not only the number of irradiated lesions.

Finally, resistance should be analyzed mechanistically rather than defined by progression alone. Paired tissue and molecular studies should determine whether failure reflects target loss, impaired delivery, altered processing, or payload resistance. Payload diversification is supported by preclinical evidence but requires prospective clinical validation before it can guide sequencing [31].

## 16. Limitations

This narrative review cannot guarantee exhaustive retrieval or estimate a pooled treatment effect. The supplementary search strategies are reconstructed formulations of the reported concepts. Incomplete access to detailed study information, described in the supplementary evidence tables, limits some schedule-specific comparisons.

The evidence spans intact ADCs, free payloads, experimental carriers, and models with different immune and species-binding constraints. Clinical reports vary in indication, concurrency, prior RT, dose, endpoints, and observation time; overlapping cohorts and clustered lesions further limit comparison.

The four-window framework has not been prospectively validated. Systemic-treatment randomization does not establish RT-timing effects, and registry or protocol information cannot substitute for outcome data.

## 17. Conclusions

ADC–RT interactions depend on the conjugate, tissue, radiation regimen, and host. Direct experiments support selected targeting, payload, and immune mechanisms, while clinical cohorts identify feasibility and organ-specific concerns rather than a universal combination benefit.

The four windows connect a mechanism to competing schedules, measurements, and harm. Their practical value should be tested through drug- and organ-specific comparisons that distinguish exposure changes from the altered response at a comparable exposure.

Progress will depend on demonstrating a durable clinical benefit with acceptable acute and delayed injury, rather than assuming that treatment overlap improves the therapeutic index.

## Figures and Tables

**Table 1:** Mechanistic evidence and priority questions for clinical translation.

Biological Domain	Representative Evidence and Class	Supported Conclusion	Priority Unresolved Question
Target modulation	Indirect target/antibody study [4]; direct TIP1–MMAE and ICAM1–DXd ADC–RT [6,17]	Some targets are radiation responsive in the studied systems	Does induction increase productive tumor exposure without increasing relevant normal tissue exposure?
Tissue access	Indirect: vascular imaging and unconjugated-antibody/probe uptake [5,24,25]	RT can modify uptake or vascular behavior under specific conditions	Does increased bulk uptake improve spatial payload distribution after clinically relevant fractionation?
Direct linker activation	Engineered cleavable carriers, not marketed ADCs [18]	Radiation-triggered release is chemically feasible	Can activation remain selective and effective under heterogeneous tissue oxygenation and dose deposition?
Payload interaction	Direct ADC–RT plus separate free-payload and peptide–RT arms [19]	Payload, linker, carrier, exposure, and endpoint jointly influence response	Which interactions persist with intact ADCs and clinically relevant exposure?
Immune contribution	Direct auristatin-conjugate/RT immune experiments [7]; T-DXd immune studies without RT [16,36]	Selected combination arms demonstrate immune contribution; no-RT studies inform mechanism only	Does RT add functional immune benefit beyond either treatment alone?
Immune counter-regulation	Indirect RT-only immune constraints: TREX1 and lymphocyte depletion [8,9]	Radiation-induced tumor damage can coexist with impaired immune function	Which schedules preserve useful immune populations and antigen presentation?
Clinical tolerability	Clinical ADC–RT observations; no randomized timing comparison [42,47,48]	Selected combinations can be delivered in the reported settings	What is the incremental toxicity attributable to treatment overlap?
Delayed intracranial injury	Clinical association and candidate astrocyte mechanism [11]; mixed-ADC association [49]	A clinically important association warrants drug-specific investigation	How do timing, lesion volume, prior RT and survival modify risk?

Direct ADC–RT denotes an intact antibody–drug conjugate combined with irradiation in the relevant experimental arm. ADC without RT and indirect component or platform evidence are identified separately. See Supplementary Table S1 for study conditions. ADC, antibody–drug conjugate; RT, radiotherapy.

**Table 2:** Payload and platform considerations for combination studies.

Payload or Platform	Rationale for Cooperation	Principal Concern	Testable Design Question
MMAE and auristatin conjugates	Microtubule disruption and preclinical radiosensitization; immune contributions in selected models [7,19]	Tumor radiosensitization may not translate into an improved therapeutic index	Does scheduling around active payload exposure improve durable control at comparable normal tissue toxicity?
DM1-containing T-DM1	Microtubule disruption and evidence supporting interaction with radiation [11,50,51]	Intracranial radionecrosis association and reported acute skin toxicity [11,50]	Which lesion, dose and timing characteristics identify excess risk?
DXd-containing T-DXd	DNA damage, bystander activity and immune modulation in experimental systems [16]	Pulmonary toxicity and uncertainty about organ-specific interaction with RT [44,52]	Can drug-related lung injury and radiation pneumonitis be distinguished through adjudication and dosimetry?
SN-38-containing sacituzumab govitecan	Potential interaction between topoisomerase-I inhibition and radiation damage	Hematologic toxicity and limited evidence for extensive marrow or bowel irradiation [45,46]	How do irradiated volume and infusion timing influence toxicity and treatment completion?
Radiation-cleavable conjugates	Direct activation through radiation-responsive chemistry [18]	Release in exposed normal tissues and uncertain distribution after cleavage	Can tumor activation be increased without proportionally increasing normal tissue exposure?
STING-agonist conjugates	Targeted innate activation could complement radiation-induced tumor damage [40,41]	Systemic inflammatory exposure and uncertain functional benefit of pathway convergence	Does RT add antigen presentation and tumor control beyond the conjugate alone?

These are research questions, not treatment recommendations. A liability belongs to the named conjugate and organ context, and cannot automatically be assigned to every agent sharing its payload class. RT, radiotherapy.

**Table 3:** Selected clinical evidence and limits of interpretation.

Study	Design and Denominator	Relevant Observation	Principal Limitation
T-DXd|TENDANCE [10]	Retrospective; 54 patients	No RT-related ADC discontinuation; median follow-up 9 months	Mixed RT regimens; short late-toxicity observation
T-DXd|COMBART [42]	Prospective observational; 40 patients/98 lesions	4 patients with late events; 21 brain/34 thoracic lesions; median follow-up 11 months	Lesion counts are not organ-specific patient denominators
T-DXd|Visani et al. [47]	147 patients: 67 RT, 80 no RT	Grade ≥3 events: 8/67 vs 16/80; median follow-up 9 months	Different baseline risk; no lung metastases irradiated
T-DXd|Khatri et al. [53]	34 patients/215 lesions/48 SRS courses	24-month symptomatic RN: 2.1% per lesion; 11% per patient	Different units; competing death and lesion clustering
T-DXd|Chun et al. [54]	113 patients; 29 T-DXd-exposed	No RN in exposed group; shorter treatment-level follow-up	Endpoint-dependent exposure; no zero-risk inference
T-DM1|Stumpf et al. [11]	45 selected patients; 23 exposed/22 unexposed	Clinically significant RN: 9/23 vs 1/22; OR 13.5 (95% CI 1.5–118.7)	Exposure not restricted to concurrent administration
Mixed ADC|Koide et al. [49]	168 patients; 19 concurrent ADC recipients	2-year symptomatic RN: 27% vs 7%	Small mixed-agent exposed group; prior RT confounding
SG|ATTENTION [48]	Retrospective; 55 patients/63 lesions	No grade ≥3 toxicity with concomitant SG; median follow-up 7.9 months	Study-defined ±4-day interval; short observation
EV/SG|Lock et al. [58]	103 patients/166 courses; 60 concurrent patients	10/60 stopped ADC for grade 3–5 toxicity; one possibly RT-influenced case	Mixed agents; investigator attribution; heterogeneous fields
Disitamab/toripalimab|Chen et al. [43]	10 patients; 6 cT1, 4 cT2–4a	9 completed RT; 2 late hemorrhagic cystitis events	Mixed stages; triplet attribution; bladder function unresolved
Polatuzumab|Melancon et al. [63]	Retrospective; 21 patients with aggressive B-cell lymphoma	Concurrent: within 3 weeks before/during RT; median follow-up 3.5 months	Abstract-based; baseline marrow toxicity, co-treatment and competing mortality
T-DXd vs T-DM1|DESTINY-Breast05 [44]	1635 randomized systemic-treatment patients	Drug-related ILD 9.6% vs 1.6%	RT timing not randomized; secondary abstract is same trial

See Supplementary Table S2 for site, dose/fractionation, timing definition, previous RT, follow-up, endpoint unit, and source limitations. Secondary analyses and pooled syntheses are not independent cohorts. ADC, antibody–drug conjugate; CI, confidence interval; EV, enfortumab vedotin; ILD, interstitial lung disease; OR, odds ratio; RN, radionecrosis; RT, radiotherapy; SG, sacituzumab govitecan; SRS, stereotactic radiosurgery. T-DM1, trastuzumab emtansine; T-DXd, trastuzumab deruxtecan.

**Table 4:** Investigational biomarkers for mechanism-based ADC and RT studies.

Biological Question	Measurement Needed to Support the Claim	Sampling Strategy	Supportive or Optional Measures and Limits
Does RT increase accessible target?	Serial membrane-accessible target assessment in the relevant model/tissue	Before treatment and at prespecified intervals around the proposed induction window	ImmunoPET and perfusion are supportive; improved delivery additionally requires actual conjugate/payload exposure
Does antibody distribution improve?	Spatial localization of the actual intact ADC in viable tumor	Baseline and mechanism-informed intervals after RT	Unconjugated-antibody PET/fluorescence and vascular imaging are surrogates; not direct payload penetration
Does active payload exposure increase?	Validated assay distinguishing intact ADC, conjugated drug, and released payload	Around infusion and selected radiation fractions	DNA-damage/mitotic markers support pharmacodynamics but are not drug-specific; comparable exposure may still permit radiosensitization
Is functional immune priming achieved?	Functional immune contribution: depletion/perturbation in models or convergent tumor-reactivity evidence clinically	Baseline, early treatment and later immune-response timepoints	Spatial profiling, T-cell receptor tracking, cytokines, and ICD markers are optional supportive assays
Are relevant lymphocyte populations preserved?	Core: serial blood counts; mechanistic claim: relevant tumor-reactive lymphocyte function	Across treatment blocks and recovery periods	Paired tissue/clonotype studies optional; blood counts alone do not establish intratumoral function
What mechanism accompanies progression?	Mechanism-matched progression sample or functional assay when resistance reversal is claimed	At progression, with comparison against baseline material	ctDNA is optional and can detect selected genomic changes; it does not measure membrane target loss or intracellular processing

ADC, antibody-drug conjugate; ctDNA, circulating *tumor* DNA; PET, positron-emission tomography; RT, radiotherapy.

**Table 5:** Core clinical reporting dataset and optional mechanistic substudies.

Dataset Tier and Domain	Information to Report
Core|Clinical setting	Disease and stage; treatment intent; eligibility; performance status; previous systemic and local treatments; reason for adding RT
Core|ADC exposure	Generic agent name; target; linker and payload; administered dose; cycle dates; dose reductions; interruptions; previous ADCs and payload classes
Core|RT exposure	Anatomical site; technique; total dose; fraction size and number; treatment dates; target volume; relevant organ dose-volume measures
Core|Temporal relationship	Interval between each ADC infusion and relevant radiation fractions; explicit definition of concurrent treatment; rationale for any interruption or washout
Core|Disease characteristics	Target-assessment method and timing; lesion size and location; measurable disease outside the radiation field; previous irradiation and reirradiation
Core|Other treatment	Checkpoint inhibitors, chemotherapy, corticosteroids and other relevant concomitant treatments, with administration dates
Core|Toxicity	Grading system and version; baseline abnormalities; attribution; onset; duration; management; recovery; acute and late observation periods
Core|Outcomes and analysis	Patient and lesion denominators; endpoint definitions; follow-up; missing data; competing events; adjustment for lesions clustered within patients
Optional|Mechanistic substudy	Select assays required by the hypothesis (Table 4); report sampling, validation, paired-sample availability, and missingness. Serial biopsies, immunoPET, tissue payload assays, and functional immune tests are not mandatory for every clinical cohort.

**Table 6:** Selected prospective studies combining ADCs and RT.

Study and Registry Identifier	Regimen and Setting	Design and Treatment Strategy	Principal Question
STAR-EV, NCT06394570 [59]	Enfortumab vedotin and stereotactic RT for localized, cisplatin-ineligible muscle-invasive bladder cancer	Phase I/II neoadjuvant study before radical cystectomy; evaluates sequential and overlapping schedules	Safety of the combined regimen and pathological complete response at cystectomy
CONSOLIDATE, NCT06434350 [60]	Enfortumab vedotin with RT for locally advanced bladder cancer	Phase I/II study of concurrent treatment	Safety and tolerability of local treatment intensification
EV-PRIME, NCT06470282 [61]	Enfortumab vedotin, pembrolizumab and RT for muscle-invasive bladder cancer	Phase Ib/II study with dose evaluation and expansion in a bladder-preserving treatment strategy	Recommended dose, toxicity and clinical complete response
PRaG3.0, NCT05115500 [65]	Disitamab vedotin, hypofractionated RT, PD-1 or PD-L1 blockade, GM-CSF and IL-2 for refractory HER2-expressing solid tumors	Single-arm phase II study; the published protocol describes sequenced ADC, radiation and immune treatment	Objective response and safety of the complete regimen
NCT07579598 [68]	Becotatug vedotin or sacituzumab tirumotecan with stereotactic RT for recurrent or metastatic salivary gland carcinoma	Exploratory phase II study; RT is introduced after specified stable-disease assessments or oligometastatic progression, followed by continuation of the original ADC	Objective response and tolerability of the treatment strategy

This selective table describes study designs rather than current recruitment status or outcomes. The reported registry cutoff is 1 October 2026. Dynamic records and later protocol publications may differ and should be identified by version and access date. PRaG3.0 is a published protocol, not an outcome report [65]. GM-CSF, granulocyte–macrophage colony-stimulating factor; IL-2, interleukin-2; PD-L1, programmed death ligand 1.

## Data Availability

No new data were created or analyzed in this study. Data sharing is not applicable to this article.

## Supplementary Materials

The following supporting information can be downloaded at: https://www.mdpi.com/article/10.3390/cancers18213554/s1. Supplementary Methods S1: Literature retrieval and verification; Supplementary Table S1: Principal mechanistic studies; Supplementary Table S2: Selected clinical studies. References [4,6,7,10,11,16,17,18,19,36,42,43,44,45,46,47,48,49,50,51,52,53,54,55,58,62,63] are cited in the supplementary materials.

## Abbreviations

ADC, antibody–drug conjugate; AQP4, aquaporin-4; ATP, adenosine triphosphate; CCL8, C-C motif chemokine ligand 8; cGAS, cyclic GMP–AMP synthase; CI, confidence interval; CNS, central nervous system; ctDNA, circulating tumor DNA; DAR, drug-to-antibody ratio; DXd, deruxtecan payload; EGFR, epidermal growth factor receptor; EV, enfortumab vedotin; GM-CSF, granulocyte–macrophage colony-stimulating factor; HER2, human epidermal growth factor receptor 2; HMGB1, high-mobility group box 1; ICAM1, intercellular adhesion molecule 1; ICD, immunogenic cell death; IL-2, interleukin-2; ILD, interstitial lung disease; MHC-I, major histocompatibility complex class I; MMAE, monomethyl auristatin E; OR, odds ratio; PD-1, programmed cell death protein 1; PD-L1, programmed death ligand 1; PET, positron emission tomography; RT, radiotherapy; SBRT, stereotactic body radiotherapy; SD, standard deviation; SG, sacituzumab govitecan; SLFN11, schlafen family member 11; SRS, stereotactic radiosurgery; STING, stimulator of interferon genes; T-DM1, trastuzumab emtansine; T-DXd, trastuzumab deruxtecan; TIP1, tax-interacting protein 1; TLR4, Toll-like receptor 4; TOP1, topoisomerase I; TREX1, three-prime repair exonuclease 1; TROP2, trophoblast cell-surface antigen 2.
