# Supplementary material for: Radiotherapy-Conditioned Antibody–Drug Conjugate Activity: Molecular Mechanisms, Therapeutic Windows and Organ-Specific Toxicity
Source: Cancers (Basel). 2026 Nov 5;18(21):3554. doi: 10.3390/cancers18213554 (PMC13761411; doi:10.3390/cancers18213554)
Supplement: Supplementary file 1 [file cancers-18-03554-s001.zip › cancers-4639025-supplementary.pdf]

Supplementary methods and study level evidence

These supplementary materials describe literature retrieval and summarize study conditions, outcomes, and limitations for the ADC–RT review. Experimental platforms and clinical cohorts are distinguished so that evidence can be interpreted in its original context.

Supplementary Methods S1 Literature retrieval and verification

The reported database and registry search cutoff was October 1, 2026. The formulations below provide executable examples of the search concepts used in this critical narrative review; they are representative strategies rather than archived search histories. Selection prioritized primary mechanistic studies and clinical reports relevant to ADC–RT interactions. The review does not use a systematic-review screening flow or calculate a pooled effect.

The representative strategies include the individual ADCs, targets, and experimental conjugates listed explicitly in the database queries below.

PubMed principal strategy

("Antibody-Drug Conjugates"[MeSH Terms] OR "antibody-drug conjugate"[Title/Abstract] OR "antibody-drug conjugates"[Title/Abstract] OR "antibody drug conjugate"[Title/Abstract] OR "antibody drug conjugates"[Title/Abstract] OR "trastuzumab emtansine"[Title/Abstract] OR "trastuzumab deruxtecan"[Title/Abstract] OR "sacituzumab govitecan"[Title/Abstract] OR "enfortumab vedotin"[Title/Abstract] OR "disitamab vedotin"[Title/Abstract] OR "polatuzumab vedotin"[Title/Abstract] OR "brentuximab vedotin"[Title/Abstract] OR "belantamab mafodotin"[Title/Abstract] OR "becotatug vedotin"[Title/Abstract] OR "sacituzumab tirumotecan"[Title/Abstract] OR "XMT-2056"[Title/Abstract] OR "TAK-500"[Title/Abstract] OR "CDX3379"[Title/Abstract] OR "ICAM1"[Title/Abstract] OR "TIP1"[Title/Abstract]) AND ("Radiotherapy"[MeSH Terms] OR radiotherap*[Title/Abstract] OR radiation[Title/Abstract] OR irradiation[Title/Abstract] OR radiosensiti*[Title/Abstract] OR radiosurgery[Title/Abstract] OR SRS[Title/Abstract] OR SBRT[Title/Abstract])

Scopus principal strategy

TITLE-ABS-KEY("antibody-drug conjugate*" OR "antibody drug conjugate*" OR "trastuzumab emtansine" OR "trastuzumab deruxtecan" OR "sacituzumab govitecan" OR "enfortumab vedotin" OR "disitamab vedotin" OR "polatuzumab vedotin" OR "brentuximab vedotin" OR "belantamab mafodotin" OR "becotatug vedotin" OR "sacituzumab tirumotecan" OR "XMT-2056" OR "TAK-500" OR "CDX3379" OR "ICAM1" OR "TIP1") AND TITLE-ABS-KEY(radiotherap* OR radiation OR irradiation OR radiosensiti* OR radiosurgery OR SRS OR SBRT)

ClinicalTrials.gov strategy

("antibody drug conjugate" OR "trastuzumab deruxtecan" OR "trastuzumab emtansine" OR "sacituzumab govitecan" OR "enfortumab vedotin" OR "disitamab vedotin" OR "becotatug vedotin" OR "sacituzumab tirumotecan") AND (radiotherapy OR radiation OR irradiation OR stereotactic)

Mechanistic extensions combine the ADC or target/payload terms with target expression, internalization, linker cleavage, payload release, DNA damage, microtubules, topoisomerase I, cGAS–STING, TREX1, immunogenic cell death, lymphocytes, pneumonitis, or radionecrosis. Studies without RT are retained for defined mechanistic relevance and labeled accordingly.

Extraction distinguishes experimental arms and controls and patient, lesion, and course units, treatment conditions, follow-up, and endpoints. Primary reports take precedence. Protocols describe planned investigations; conference abstracts and secondary analyses are identified separately.

Cohort overlap: Loibl [44] and Untch [52] report the same DESTINY-Breast05 trial. Salvestrini [51] and Zare [55] synthesize existing cohorts. Institution, recruitment dates, eligibility, and trial identifier inform overlap assessment; no pooled patient total is calculated.

Supplementary Table S1 summarizes the principal mechanistic studies and distinguishes direct ADC–RT experiments from indirect evidence.

Supplementary Table S1 Principal mechanistic studies

Study	Evidence and model	Conjugate and dose	RT and timing	Controls and functional endpoint	Exposure and interpretation
Lewis et al. [6]	Direct intact ADC–RT; A549/H1299 lung xenografts, immune-deficient hosts	TIP1; 7H5–VcMMAE; 100 µg IV on days 1 and 3	2 Gy × 5; A549 days 2–6, H1299 days 1–5. Cell experiments: 48-hour ADC exposure before irradiation.	ADC alone, RT alone, vehicle, and unconjugated-antibody controls; clonogenic response and established-tumor growth	Antibody localization/internalization supports targeting; no direct released-MMAE quantification established by the examined experiments. Model-specific schedules cannot define a clinical interval.
Hingorani et al. [7]	Direct ADC–RT and separate peptide–RT arms; B16/LL2/MC38 immunocompetent models	HER3-directed CDX3379–MMAE and activatable cell-penetrating peptide–MMAE; 2.5 nmol ADC versus 10 nmol peptide corresponds to 10 nmol MMAE for DAR 4 versus 1	Selected B16/LL2 experiments: 5 Gy × 3; MC38 peptide experiments: 3 Gy × 3. Regimen depends on arm; do not transfer one schedule to all models.	Individual-treatment controls, CD8 depletion, rechallenge, and checkpoint-blockade experiments; durable tumor control and immune dependence	Carrier-associated fluorescence is an exposure surrogate. Antibody and peptide experiments have different activation and distribution; immune findings must be assigned to the actual arm.
Xing et al. [17]	Direct intact ADC–RT; HCT116 and SUM190 xenografts in nude mice	Human ICAM1–DXd; drug-to-antibody ratio approximately 8; efficacy dose 5 mg/kg	Efficacy: 2 Gy × 5. In vitro 2-Gy induction began by 12 hours and approached a 48-hour plateau; HCT116 expression persisted beyond one week. Antibody-uptake arm: antibody 24 hours after 2 Gy. Exact optimal efficacy interval not established.	Vehicle, RT, ADC, combination; established-tumor growth. Induction varied by cell model; SUM190 responded to fractionated 2 Gy × 3.	Fluorescent unconjugated antibody, not quantified intact ADC/released DXd. Antibody lacks mouse ICAM1 cross-reactivity; nude hosts limit adaptive-immune and normal-tissue inference.
Lesperance et al. [19]	Direct ADC–RT in established B16 tumors in immunocompetent mice; separate peptide and free-payload/implantation arms	HER3-directed CDX3379–MMAE versus CDX3379–DXd; matched DAR 4, different linkers; 2.5 nmol IV per ADC dose	Two doses 48 hours apart; 5 Gy × 3 beginning the day after the first dose. Separate free-payload cell exposures preceded irradiation and implantation.	ADC, RT, combination, and control arms; established-tumor control. MMAE ADC improved control versus RT alone under tested conditions; DXd ADC did not significantly improve this comparison.	Carrier imaging is not released-payload exposure. Free-payload implantation tests tumor establishment; peptide immune profiling is a separate arm. Target/DAR matching does not eliminate linker confounding.

Study	Evidence and model	Conjugate and dose	RT and timing	Controls and functional endpoint	Exposure and interpretation
Quintana et al. [18]	Indirect engineered radiation-cleavable platforms, including non-antibody carriers; chemical and cell experiments	Doxorubicin/MMAE with radiation-responsive linker chemistry	Cell-free release included 8-Gy irradiation; oxygen conditions altered activation. Some cell assays used conjugates irradiated before exposure.	Unirradiated/irradiated conjugates and free-drug controls; measured chemical release and cytotoxicity	Direct chemical release measurement supports cleavage. Preirradiation and non-antibody carriers do not establish in vivo activation of marketed ADCs by external-beam RT.
Wattenberg et al. [4]	Indirect; irradiated tumor cells and unconjugated antibody; no ADC payload	Selected HER2, EGFR, CD20 targets; trastuzumab functional assays	Single 5 or 10 Gy; surface-target assays at 48 hours. MCF-7 HER2 was followed at 24–96 hours. ADCC assays used antibodies 48 hours after RT (20 µg/mL, 30-minute binding), followed by a 4-hour effector-cell assay.	Surface-antigen assessment and antibody-dependent cellular cytotoxicity	Target and antibody-function measurements are not intact-ADC distribution or payload delivery.
Tsao et al. [16]; Iwata et al. [36]	Intact T-DXd without RT; breast-cancer and immune mouse systems	HER2-directed T-DXd; protease-dependent release and immune effects	No RT; no drug–RT interval can be extracted	Protease perturbation/myeloid signaling (Tsao); PD-1 combination and immune response (Iwata)	Supports ADC biology, not an incremental radiation effect. Do not group these as direct ADC–RT immune experiments.
Stumpf et al. [11]	Direct T-DM1–irradiation astrocyte experiments, alongside a clinical SRS association	Human astrocyte system; T-DM1 versus trastuzumab, 1 µg/mL	0, 2, 4, or 8 Gy; AQP4 assessment at 48 hours and confluence/swelling over several days	Drug/irradiation comparators; AQP4 dysregulation and astrocytic swelling; limited patient pathology	Candidate tissue-injury mechanism; no universal human causal mechanism, clinical biomarker threshold, or validated holding interval.

Supplementary Table S2 expands manuscript Table 3 with treatment conditions and outcome denominators. Doses are expressed as total Gy/number of fractions unless another unit is specified. See the source-scope and interpretation notes below the table.

Supplementary Table S2 Selected clinical studies

ADC and study	Population and unit	RT exposure and concurrency	Follow-up and outcomes	Interpretation and source limitations
T-DXd | Debbi et al. [10], TENDANCE	Retrospective; 54 patients with metastatic breast cancer; palliative and ablative sites	RT during ongoing T-DXd. Schedules included 8 Gy/1, 20 Gy/5, 30 Gy/10, 21 Gy/3, and 27 Gy/3. Median last infusion to RT: 10 days (1–21); RT completion to next infusion: 11 days (3–25). Prior-RT distribution is not given in the main report.	Median follow-up 9 months from RT completion. No RT-related T-DXd discontinuation or dose reduction. Three grade 1 radionecrosis events after SRS; three grade 1 ILD events.	Whole-cohort percentages do not estimate organ-specific risk. Short observation limits delayed-toxicity assessment; uncontrolled response cannot establish an RT interaction.
T-DXd | Ippolito et al. [42], COMBART	Prospective observational; 40 patients, 98 lesions; 21 brain and 34 thoracic lesions, including 5 lung lesions	RT on infusion day or within the 21-day intercycle interval. Heterogeneous fractionation. The original dose table expresses total dose/dose per fraction, rather than total dose/number of fractions. Individual previous-RT histories require case-level interpretation.	Median follow-up 11 months (6–31). Four patients had late events: 3 pneumonitis and 3 radionecrosis events, including grade 3 radionecrosis at 16 months. Among 22 oligoprogression patients, median time to systemic-treatment change was 19.1 months (95% CI 12.7–25.56).	Lesion counts cannot supply organ-specific patient denominators. Several events occurred in the same patients. Treatment continuity was observed without a randomized comparator.
T-DXd | Visani et al. [47]	Retrospective; 147 patients: 67 RT recipients with 71 courses, 80 without RT; 39/71 courses involved the CNS	RT during T-DXd or within 10 days of its first dose. Median total dose 44 Gy (8–48); median 4 fractions (1–20). These separate medians do not define one regimen. No lung metastases were irradiated. Prior thoracic RT was listed among collected variables, but no distribution is tabulated in the main report.	Median follow-up 9 months (0.5–46). Grade ≥3 events: 8/67 versus 16/80 (p = 0.30). Toxicity-related T-DXd discontinuation: 8/67 versus 11/80.	RT recipients were younger and more often had CNS disease. The nonsignificant comparison does not establish equivalence; the study cannot establish safety of irradiation of lung metastases.
T-DXd | Khatri et al. [53]	Retrospective; 34 patients, 215 brain lesions, 48 SRS courses; 19/34 patients had previous intracranial RT	Single-fraction SRS: median 22 Gy (15–24); fractionated SRS: median 24 Gy (15–35) in median 3 fractions (3–5). Eligibility permitted SRS within 3 months before T-DXd initiation or after discontinuation. Concurrent SRS occurred during treatment or ≤1 month after the last infusion; 117/215 lesions met this definition.	Median follow-up: 13.9 months (0.9–31.8) from T-DXd initiation, 11.8 months (0.1–30.2) per SRS course. At 24 months, symptomatic radionecrosis was 2.1% per lesion and 11% per patient. Local control: 97% at 12 months (95% CI 93–99) and 88% at 24 months (75–94).	Death was a competing event for radionecrosis. Patient and lesion estimates differ; small targets, previous RT, and repeated lesions limit generalization. No RT-alone comparator establishes incremental benefit.

ADC and study	Population and unit	RT exposure and concurrency	Follow-up and outcomes	Interpretation and source limitations
T-DXd | Chun et al. [54]	Retrospective; 113 patients, 29 T-DXd-exposed; 461 SRS treatments; previous whole-brain RT in 10/29 exposed patients	Concurrent window within 3 months. Endpoint-dependent exposure assignment: 137 T-DXd-positive treatments for safety versus 61 for efficacy. Fractionation distributions differed between groups.	No radionecrosis in the 29 exposed patients. Mean treatment follow-up: 15.5 versus 28.1 months. Mean patient follow-up overall: 28.7 months (SD 23.3).	Unequal observation and endpoint-dependent exposure definitions limit direct comparisons. Zero observed events do not establish zero risk or a protective effect.
T-DM1 | Stumpf et al. [11]	Retrospective selected cohort; 45 patients: 23 with T-DM1 exposure, 22 without	Brain SRS in up to 5 fractions. Sixteen exposed patients received concurrent treatment within 4 weeks. The principal comparison uses any T-DM1 exposure. Fifteen of 45 patients received whole-brain RT at some point in their intracranial treatment course.	Clinically significant radionecrosis: 9/23 versus 1/22; odds ratio 13.5 (95% CI 1.5–118.7). Median SRS-to-radionecrosis latency was 16 months (1–79); this is not cohort follow-up. Proportions are not fixed 2-year cumulative-incidence estimates.	Age/HER2 selection, previous or repeated irradiation, and broad drug-exposure histories limit generalization. An odds ratio is not a risk ratio; the association does not establish an optimal holding interval.
Mixed ADCs | Koide et al. [49][1]	Retrospective; 168 patients, including 19 concurrent ADC recipients; 33% had previous brain RT	Concurrent ADC: within 4 weeks before or after brain RT. The primary abstract does not specify dose/fractionation; it therefore cannot support a regimen-specific comparison.	Median follow-up 31 months. Symptomatic radionecrosis: 5/19 versus 13/149; 2-year cumulative incidence 27% versus 7%. Adjusted subdistribution hazard ratio 3.0 (95% CI 1.1–8.3), treating death as a competing event.	Small mixed-agent exposed group; the findings do not provide a T-DXd-specific absolute risk. Previous RT and fractionation need stratification before cross-study comparison.
T-DM1 | Dastgheyb et al. [50]	Retrospective; 35 patients receiving breast/chest-wall RT with or without regional nodes	T-DM1 within 14 days of RT initiation. Median total dose 50 Gy (30–60.4), across 10–33 fractions. First RT course in 32/35; no overlapping reirradiation. Techniques: 28 three-dimensional conformal, 5 proton, 2 IMRT/VMAT.	Median follow-up 20 months. Three patients had grade 3 dermatitis; no grade 4–5 skin toxicity or persistent dermatologic complaints were reported. Grade ≥2 counts differ between the abstract and main results and are not combined here.	The unambiguous grade 3 numerator is 3/35. The abstract states 22/35 grade ≥2, whereas the results/table give 23 grade 2 plus 3 grade 3. Acute dermatitis cannot estimate delayed CNS or lung injury.
SG | Krug et al. [45]	Retrospective six-center cohort; 17 patients, 34 palliative RT courses; 12 patients had previous RT	Eligibility: SG within 90 days before/after RT. Median total dose 36 Gy (8–50.4), median dose per fraction 3 Gy (1.8–20). Twelve courses overlapped previous fields; median interval 2 years (1–7). Sequential treatment: median SG-to-RT gap 7 days (1–33), resumption 6 days (1–68).	Three in-field grade 3 events (2 dermatitis, 1 esophagitis), all with previous-field overlap; no grade 4–5 acute/late toxicity reported. RT response assessed at 4–8 weeks. Median survival after RT was 107 days; this is not median follow-up.	The table lists 7 simultaneous patients, while the narrative lists 8; no simultaneous-subgroup rate is reproduced. Small cohort, repeated courses, and short survival limit delayed-risk assessment.

ADC and study	Population and unit	RT exposure and concurrency	Follow-up and outcomes	Interpretation and source limitations
SG | Loap et al. [46][1]	Retrospective single-center cohort; 13 patients; 19 irradiated metastatic sites: 10 brain, 9 bone	Concurrent SG and external-beam RT. Exact dose/fractionation, concurrency boundary, and previous-RT distribution are not stated in the primary abstract.	No radiation-induced toxicity or treatment interruption reported. Grade 3–4 neutropenia occurred in 15.4%. Median survival from RT completion was 6 months; this is not a follow-up estimate.	Abstract-supported description only. Small sample and competing mortality cannot establish absence of delayed toxicity; field-specific risk requires the full treatment details.
SG | Alati et al. [48], ATTENTION[1]	Retrospective six-center cohort; 55 patients, 63 lesions; 37 concurrent and 26 sequential lesions; bone 49%, brain 32%	Concurrent RT: within 4 days before/after SG; median interval 3 days versus 10 days sequentially. Three-dimensional conformal RT 52%, SBRT 40%, IMRT 8%. Exact fractionation and previous RT are not stated in the primary abstract.	Median follow-up 7.9 months (1.7–35.4). No grade ≥3 toxicity with concomitant SG. Response assessment at 4–12 weeks after RT.	Lesion-based exposure groups are not patient denominators. The abstract's complete/partial-response percentages total >100% and are not reproduced. The observed ±4-day definition is not a validated safe interval.
EV or SG | Lock et al. [58]	Retrospective two-center cohort; 103 patients, 166 courses. Concurrent subgroup: 60 patients, 77 courses	Eligibility: RT within 6 months of ADC. Concurrent: RT initiation within 4 weeks before/after infusion. RT included 8 Gy/1 and 20 Gy/5 palliative courses and ablative schedules such as 40 Gy/5. Previous-field overlap is described in individual cases rather than a complete reirradiation stratum.	Ten of 60 concurrent patients stopped ADC for grade 3–5 toxicity. One possibly RT-influenced case followed SG and craniospinal RT (30 Gy/15 plus 8 Gy/2 boost), with neutropenia and fatal pneumonia. A cohort-wide median follow-up is not supplied in the main report.	Toxicity attribution does not remove the total event burden. Use the patient denominator (10/60), rather than dividing patients by courses. Mixed drugs, repeated courses, and large fields limit extrapolation to curative settings.
Disitamab vedotin/toripalimab | Chen et al. [43]	Ten patients: 6 cT1, 1 cT2, 2 cT3, 1 cT4a; bladder-preserving treatment	Disitamab 2 mg/kg plus toripalimab 240 mg every 2 weeks. Planned bladder/pelvic RT: 60–64 Gy at 2 Gy/fraction or 55 Gy/20. One patient stopped at 46 Gy for personal reasons. A previous-RT distribution is not supplied in the main report.	Median follow-up 17.2 months (6–31). Nine completed RT; one grade 3 leukopenia. Grade 1–2 cystitis in 9; delayed hemorrhagic cystitis at 20 and 22 months. Nine retained the bladder.	Mixed non-muscle-invasive and muscle-invasive disease without a comparator. Organ retention does not establish preserved function. Internally inconsistent survival confidence-interval bounds are not reproduced.

ADC and study	Population and unit	RT exposure and concurrency	Follow-up and outcomes	Interpretation and source limitations
Disitamab vedotin/toripalimab | Zhang et al. [62]	Six-patient first stage of a single-center phase II study, NCT05979740; localized HER2-positive muscle-invasive bladder cancer	After maximal transurethral resection: disitamab 2 mg/kg plus toripalimab 3 mg/kg every 2 weeks. RT started in cycle 2 or 3, at 2 Gy/fraction: initial bladder ± pelvic phase 46 Gy/23 followed by a bladder boost. A previous-RT distribution is not supplied in the main report.	Median follow-up 12.3 months (10.7–12.8). Five of six had complete response and remained recurrence-free. Two had grade 3 treatment-related events; one stopped drugs for liver injury. No grade 4 treatment-related events or treatment-related deaths. Urinary tumor DNA monitoring was exploratory.	Small uncontrolled first-stage cohort; clinical benefit and biomarker utility require validation. The report's boost wording is ambiguous, so no additional boost dose is inferred. This is a separate report from Chen.
Polatuzumab vedotin | Melancon et al. [63][1]	Retrospective; 21 patients with aggressive B-cell lymphoma; median 4 previous systemic-treatment lines; 48% received additional concurrent systemic therapy	Polatuzumab within 3 weeks before/during RT. Acute assessment: RT start through 1 month afterward; subacute: months 2–3. Dose/fractionation and previous-RT details are not stated in the primary abstract.	Median follow-up 3.5 months; 86% deceased. Among survivors, median follow-up 20 months (IQR 7–39). Acute grade ≥3 hematologic toxicity was associated with pre-existing severe hematologic toxicity; increased acute toxicity grade was associated with RT dose.	Baseline marrow toxicity, additional systemic therapy, and competing mortality complicate attribution. Longer survivor follow-up cannot substitute for observation of the entire cohort.
T-DXd versus T-DM1 | Loibl et al. [44], DESTINY-Breast05	1,635 patients randomized to systemic therapy after high-risk residual HER2-positive early breast cancer	Systemic ADC assignment was randomized; RT timing was not. This row provides systemic pulmonary-safety context and does not assign a dose-specific RT risk.	Adjudicated drug-related ILD: 9.6% with T-DXd versus 1.6% with T-DM1. These are drug-related ILD outcomes, rather than radiation-pneumonitis incidence estimates.	The Untch et al. [52] conference analysis belongs to the same trial and is not an independent cohort. An arm-level ILD difference does not establish an ADC–RT interaction.

Doses are expressed as total Gy/number of fractions unless another unit is specified. Only source-supported treatment conditions and outcomes are tabulated. [1] Koide [49], Loap [46], Alati [48], and Melancon [63] rows use primary abstracts; details absent from those abstracts are identified explicitly and are not treated as absent from the full publications. Missing distributions in examined main reports are described with that scope. Survival is distinguished from follow-up. Reported numerical discrepancies are retained as limitations; no pooled estimate or overall patient total is calculated.

Abbreviations: ADC, antibody–drug conjugate; ADCC, antibody-dependent cellular cytotoxicity; AQP4, aquaporin-4; CD8, cluster of differentiation 8; CI, confidence interval; CNS, central nervous system; DAR, drug-to-antibody ratio; DXd, deruxtecan payload; EV, enfortumab vedotin; HER2, human epidermal growth factor receptor 2; HER3, human epidermal growth factor receptor 3; ICAM1, intercellular adhesion molecule 1; ILD, interstitial lung disease; IMRT, intensity-modulated radiation therapy; IQR, interquartile range; IV, intravenous; MMAE, monomethyl auristatin E; PD-1, programmed cell death protein 1; RT, radiotherapy; SBRT, stereotactic body radiotherapy; SD, standard deviation; SG, sacituzumab govitecan; SRS, stereotactic radiosurgery; T-DM1, trastuzumab emtansine; T-DXd, trastuzumab deruxtecan; TIP1, tax-interacting protein 1; VMAT, volumetric modulated arc therapy.

References

Reference numbers correspond to those in the main manuscript.

[4] Wattenberg, M.M.; Kwilas, A.R.; Gameiro, S.R.; Dicker, A.P.; Hodge, J.W. Expanding the use of monoclonal antibody therapy of cancer by using ionising radiation to upregulate antibody targets. Br. J. Cancer. 2014, 110, 1472–1480. https://doi.org/10.1038/bjc.2014.79. PMID: 24556625.

[6] Lewis, C.D.; Singh, A.K.; Hsu, F.F.; Thotala, D.; Hallahan, D.E.; Kapoor, V. Targeting a Radiosensitizing Antibody-Drug Conjugate to a Radiation-Inducible Antigen. Clin. Cancer Res. 2021, 27, 3224–3233. https://doi.org/10.1158/1078-0432.CCR-20-1725. PMID: 34074654.

[7] Hingorani, D.V.; Allevato, M.M.; Camargo, M.F.; Lesperance, J.; Quraishi, M.A.; Aguilera, J.; Franiak-Pietryga, I.; Scanderberg, D.J.; Wang, Z.; Molinolo, A.A.; et al. Monomethyl auristatin antibody and peptide drug conjugates for trimodal cancer chemo-radio-immunotherapy. Nat. Commun. 2022, 13, 3869. https://doi.org/10.1038/s41467-022-31601-z. PMID: 35790753.

[10] Debbi, K.; Benderra, M.A.; Medioni, J.; Durdux, C.; To, N.H.; Boukhobza, C.; Grellier, N.; Benmaziane, A.; Monnier, L.; Gligorov, J.; et al. Safety and efficacy of combined trastuzumab-deruxtecan and concurrent radiation therapy in breast cancer. The TENDANCE multicentric French study. Breast 2025, 80, 104421. https://doi.org/10.1016/j.breast.2025.104421. PMID: 39983437.

[11] Stumpf, P.K.; Cittelly, D.M.; Robin, T.P.; Carlson, J.A.; Stuhr, K.A.; Contreras-Zarate, M.J.; Lai, S.; Ormond, D.R.; Rusthoven, C.G.; Gaspar, L.E.; et al. Combination of Trastuzumab Emtansine and Stereotactic Radiosurgery Results in High Rates of Clinically Significant Radionecrosis and Dysregulation of Aquaporin-4. Clin. Cancer Res. 2019, 25, 3946–3953. https://doi.org/10.1158/1078-0432.CCR-18-2851. PMID: 30940654.

[16] Tsao, L.C.; Wang, J.S.; Ma, X.; Sodhi, S.; Ragusa, J.V.; Liu, B.; McBane, J.; Wang, T.; Wei, J.; Liu, C.X.; et al. Effective extracellular payload release and immunomodulatory interactions govern the therapeutic effect of trastuzumab deruxtecan (T-DXd). Nat. Commun. 2025, 16, 3167. https://doi.org/10.1038/s41467-025-58266-8. PMID: 40175391.

[17] Xing, Y.; Yang, Y.; Liu, Y.; Yang, T.; Ma, L.; Chen, J.; Yang, Y.; Xu, Y.; Lu, Y.; Guo, P.; et al. Low dose radiotherapy-induced tumor-preferential antigens enhance antibody-drug conjugate efficacy in solid tumors. Neoplasia 2026, 79, 101335. https://doi.org/10.1016/j.neo.2026.101335. PMID: 42476099.

[18] Quintana, J.M.; Arboleda, D.; Hu, H.; Scott, E.; Luthria, G.; Pai, S.; Parangi, S.; Weissleder, R.; Miller, M.A. Radiation Cleaved Drug-Conjugate Linkers Enable Local Payload Release. Bioconjugate Chem. 2022, 33, 1474–1484. https://doi.org/10.1021/acs.bioconjchem.2c00174. PMID: 35833631.

[19] Lesperance, J.; Yung, B.S.; Allevato, M.M.; Cheng, M.M.; Camargo, M.F.; Saddawi-Konefka, R.; Dhawan, K.; Sharma, A.K.; Mortaja, M.; Bice, S.; et al. Interactions of Antibody Drug Conjugate Anti-Tubulin and Topoisomerase I Inhibitor Payloads with Radiotherapy to Potentiate Immunotherapy. Adv. Sci. 2026, 13, e06552. https://doi.org/10.1002/advs.202506552. PMID: 41133986.

[36] Iwata, T.N.; Ishii, C.; Ishida, S.; Ogitani, Y.; Wada, T.; Agatsuma, T. A HER2-Targeting Antibody-Drug Conjugate, Trastuzumab Deruxtecan (DS-8201a), Enhances Antitumor Immunity in a Mouse Model. Mol. Cancer Ther. 2018, 17, 1494–1503. https://doi.org/10.1158/1535-7163.MCT-17-0749. PMID: 29703841.

[42] Ippolito, E.; Benincasa, M.; Pantano, F.; Greco, C.; Donato, M.; Gullotta, C.M.; Toppi, L.; Alaimo, R.; Martucci, P.; Meffe, G.; et al. Trastuzumab-deruxtecan and radiotherapy: A safety sub-analysis of COMBART prospective cohort study. Oncologist 2025, 30, oyaf229. https://doi.org/10.1093/oncolo/oyaf229. PMID: 40704702.

[43] Chen, H.; Liu, N.; Chen, X.; Xian, P.; Li, J.; Dai, J.; Yuan, G.; Yuan, F. Safety and preliminary efficacy of Disitamab Vedotin combined with Toripalimab and concurrent radiotherapy in bladder-preserving treatment for HER2-positive invasive bladder cancer. Front. Oncol. 2026, 16, 1880457. https://doi.org/10.3389/fonc.2026.1880457. PMID: 42459305.

[44] Loibl, S.; Park, Y.H.; Shao, Z.; Huang, C.S.; Barrios, C.; Abraham, J.; Prat, A.; Niikura, N.; Im, S.A.; Li, W.; et al. Trastuzumab Deruxtecan in Residual HER2-Positive Early Breast Cancer. N. Engl. J. Med. 2026, 394, 845–857. https://doi.org/10.1056/NEJMoa2514661.

[45] Krug, D.; Tio, J.; Abaci, A.; Beurer, B.; Brügge, S.; Elsayad, K.; Meixner, E.; Park-Simon, T.W.; Smetanay, K.; Winkelmann, F.; et al. The Safety and Efficacy of the Combination of Sacituzumab Govitecan and Palliative Radiotherapy-A Retrospective Multi-Center Cohort Study. Cancers 2024, 16, 1649. https://doi.org/10.3390/cancers16091649. PMID: 38730602.

[46] Loap, P.; Chabli, S.; Cottu, P.; Kirova, Y. Safety and Tolerability of Concurrent Radiotherapy and Sacituzumab Govitecan in Metastatic Breast Cancer. Am. J. Clin. Oncol. 2025, 48, 399–402. https://doi.org/10.1097/COC.0000000000001195. PMID: 40178882.

[47] Visani, L.; Ratosa, I.; Bartsch, R.; Linderholm, B.; Griguolo, G.; Saieva, C.; Becherini, C.; Valzano, M.; Salvestrini, V.; Starzer, A.M.; et al. Concomitant radiation therapy and trastuzumab deruxtecan in metastatic breast cancer: Feasibility, safety, and outcomes from a real-life multicentre international cohort. Breast 2026, 85, 104691. https://doi.org/10.1016/j.breast.2026.104691. PMID: 41496431.

[48] Alati, A.; Debbi, K.; Scher, N.; Meynard, C.; Mavrikios, A.; Huguet, F.; Boudabous, H.; Medioni, J.; Gligorov, J.; Bennassi, A.; et al. Multicenter Retrospective Analysis of the Safety and Efficacy of Sacituzumab Govitecan Combined with Radiation Therapy: The French ATTENTION Study. Int. J. Radiat. Oncol. Biol. Phys. 2026, 125, 755–762. https://doi.org/10.1016/j.ijrobp.2025.11.060. PMID: 41391611.

[49] Koide, Y.; Nagai, N.; Adachi, S.; Ito, M.; Kawamura, M.; Ito, M.; Ito, F.; Shindo, Y.; Aoyama, T.; Shimizu, H.; et al. Impact of concurrent antibody-drug conjugates and radiotherapy on symptomatic radiation necrosis in breast cancer patients with brain metastases: A multicenter retrospective study. J. Neurooncol. 2024, 168, 415–423. https://doi.org/10.1007/s11060-024-04681-8. PMID: 38644464.

[50] Dastgheyb, S.S.; Kim, K.; Doucette, A.; Freedman, G.; Shah, P.; Makhlin, I.; Clark, A.; Taunk, N. Acute skin radiation toxicity seen with concurrent T-DM1: A single institutional report of 35 patients. Breast 2023, 67, 26–29. https://doi.org/10.1016/j.breast.2022.12.002. PMID: 36566691.

[51] Salvestrini, V.; Kim, K.; Caini, S.; Alkner, S.; Ekholm, M.; Skyttä, T.; Becherini, C.; Coles, C.E.; Kaidar-Person, O.; Offersen, B.; et al. Safety profile of trastuzumab-emtansine (T-DM1) with concurrent radiation therapy: A systematic review and meta-analysis. Radiother. Oncol. 2023, 186, 109805. https://doi.org/10.1016/j.radonc.2023.109805. PMID: 37437610.

[52] Untch, M.; Shao, Z.; Huang, C.S.; Barrios, C.H.; Abraham, J.; Prat, A.; Niikura, N.; Loibl, S.; Geyer, C.E.; Geng, C.; et al. Secondary safety analysis of trastuzumab deruxtecan (T-DXd) vs trastuzumab emtansine (T-DM1) in DESTINY-Breast05: Clinical and demographic risk factors of interstitial lung disease (ILD) and radiation pneumonitis (RP). J. Clin. Oncol. 2026, 44, 516. https://doi.org/10.1200/JCO.2026.44.16_suppl.516.

[53] Khatri, V.M.; Mestres-Villanueva, M.A.; Yarlagadda, S.; Doniparthi, A.; Smith, D.B.; Nakashima, J.Y.; Bryant, J.M.; Zhao, D.; Upadhyay, R.; Mills, M.N.; et al. Multi-institutional report of trastuzumab deruxtecan and stereotactic radiosurgery for HER2 positive and HER2-low breast cancer brain metastases. NPJ Breast Cancer 2024, 10, 100. https://doi.org/10.1038/s41523-024-00711-w. PMID: 39572568.

[54] Chun, S.J.; Kim, K.; Chang, W.I.; Kim, Y.B.; Paek, S.H.; Lee, K.H.; Song, J.H.; Hong, J.H.; Lee, J.; Jang, W.I.; et al. Impact of trastuzumab deruxtecan (T-DXd) and brain stereotactic radiosurgery on intracranial control and radionecrosis risk in HER2-positive or -low breast cancer brain metastases. Breast 2026, 87, 104751. https://doi.org/10.1016/j.breast.2026.104751. PMID: 41875806.

[55] Zare, A.; Zare, A.; Kiani, I.; Delbari, P.; Mortezaei, A.; Ahmadvand, M.H.; Rashidi, F.; Mohammadzadeh, I.; Azarmi, E.; Ghalehtaki, R.; et al. Timing matters: Radiation necrosis risk of antibody-drug conjugates (ADCs) combined with radiation therapy in breast cancer brain metastases: A systematic review and comparative meta-analysis. Neurosurg. Rev. 2026, 49, 252. https://doi.org/10.1007/s10143-025-04056-1. PMID: 41748827.

[58] Lock, D.; Soni, Y.S.; Hwang, L.; Cole, S.; D’Souza, A.; Garant, A.; Yang, D.X.; Arafat, W.; Zhang, T.; Ballas, L.K.; et al. Safety of Combining Radiation Therapy and Antibody Drug Conjugates in Advanced Urothelial and Other Cancers. Adv. Radiat. Oncol. 2026, 11, 101926. https://doi.org/10.1016/j.adro.2025.101926. PMID: 41362410.

[62] Zhang, L.; Jin, D.; Zang, J.; Qian, L.; Zhang, T.; Wu, Y.; Ding, Y.; Xie, F.; Tang, H.; Xia, J.; et al. Disitamab vedotin combined with toripalimab and radiotherapy for multimodal organ-sparing treatment of muscle invasive bladder cancer: A proof-of-concept study. Neoplasia 2025, 68, 101216. https://doi.org/10.1016/j.neo.2025.101216. PMID: 40774226.

[63] Melancon, S.T.; Wang, E.B.; Fang, P.Q.; Lin, T.A.; Saifi, O.; Dabaja, B.S.; Pinnix, C.C.; Nze, C.; Nair, R.; Ahmed, S.; et al. Safety of concurrent radiation therapy with the antibody-drug conjugate polatuzumab vedotin in relapsed or refractory aggressive B-cell lymphomas. Pract. Radiat. Oncol. 2026, advance online publication (10 July 2026). https://doi.org/10.1016/j.prro.2026.06.011. PMID: 42431370.
